# Viroids and Retrozymes: Plant Circular RNAs Capable of Autonomous Replication

**DOI:** 10.3390/plants14010061

**Published:** 2024-12-27

**Authors:** Alexander A. Lezzhov, Anastasia K. Atabekova, Denis A. Chergintsev, Ekaterina A. Lazareva, Andrey G. Solovyev, Sergey Y. Morozov

**Affiliations:** A. N. Belozersky Institute of Physico-Chemical Biology, Moscow State University, 119992 Moscow, Russia; lezzhov-genetic@mail.ru (A.A.L.); asya_atabekova@mail.ru (A.K.A.); ledumpalustre86@gmail.com (D.A.C.); lazareva-katrina@mail.ru (E.A.L.); solovyev@belozersky.msu.ru (A.G.S.)

**Keywords:** long non-coding RNA, circRNA, viroids, RNA-RNA replication, retrozymes, ribozymes, plant biotechnology, circRNA biotechnology

## Abstract

Among the long non-coding RNAs that are currently recognized as important regulatory molecules influencing a plethora of processes in eukaryotic cells, circular RNAs (circRNAs) represent a distinct class of RNAs that are predominantly produced by back-splicing of pre-mRNA. The most studied regulatory mechanisms involving circRNAs are acting as miRNA sponges, forming R-loops with genomic DNA, and encoding functional proteins. In addition to circRNAs generated by back-splicing, two types of circRNAs capable of autonomous RNA-RNA replication and systemic transport have been described in plants: viroids, which are infectious RNAs that cause a number of plant diseases, and retrozymes, which are transcripts of retrotransposon genomic loci that are capable of circularization due to ribozymes. Based on a number of common features, viroids and retrozymes are considered to be evolutionarily related. Here, we provide an overview of the biogenesis mechanisms and regulatory functions of non-replicating circRNAs produced by back-splicing and further discuss in detail the currently available data on viroids and retrozymes, focusing on their structural features, replication mechanisms, interaction with cellular components, and transport in plants. In addition, biotechnological approaches involving replication-capable plant circRNAs are discussed, as well as their potential applications in research and agriculture.

## 1. Introduction

The results of extensive transcriptomic studies over the last decades have led to the understanding that long non-coding RNAs represent a substantial fraction of the transcripts produced in eukaryotic cells [1]. Functional studies indicate that these RNAs are not the result of erroneous or misregulated transcription but rather are essential regulatory molecules involved in a variety of processes in living cells [2,3]. A distinct class of long non-coding RNAs are circular RNAs (circRNAs), which are most often produced by back-splicing of pre-mRNA [4,5]. Plant viroids can be considered the first identified example of a circRNA [6,7]. Although circRNAs were discovered more than half a century ago, the biological functions of only a small number of the plant and animal circRNAs have been investigated. Cellular circRNAs are capable of protein and RNA binding, which determines their ability to regulate cell processes by depleting an active pool of circRNA-binding molecules and thereby interfering with their functions [2,8]. Moreover, being covalently closed structures that avoid degradation by exonucleases and therefore appear to be more stable in cells, circRNAs are considered as a prospective tool in biotechnological applications [9,10]. Attention to the potential use of circRNAs has recently been enhanced when circRNAs were shown to have protein coding ability [11], which would greatly expand the area of potential circRNA applications as they could serve as more stable and durable templates for protein expression in different systems.

Recently, a new class of covalently closed circRNAs called retrozymes, which are encoded by eukaryotic genomes and capable of replication, has been discovered [12]. These unique genetic elements, first described in plants, have a dual nature: in their DNA form, retrozymes are non-autonomous retrotransposons integrated into plant genomic DNA; on the other hand, in their RNA form, i.e., as transcripts of retrozyme genomic loci, they are capable of both circularization due to the presence of ribozymes and RNA-RNA replication in plant cells [12,13]. These and other features of retrozyme-specific RNAs draw parallels to viroids, infectious circRNAs that cause diseases in plants [14]; however, viroids lack their plant genome-integrated DNA counterparts. Given the structural and functional parallels between retrozymes and viroids, an evolutionary link between these two types of plant circRNAs has been proposed [14,15]. While both retrozymes and viroids are considered as non-coding RNAs, recent studies indicate that such RNAs may interact with the cell translation machinery and potentially encode protein/peptide products [16,17].

In this review, after a brief overview of the origin and functions of non-replicating plant circRNAs, we describe two known types of plant circRNAs capable of RNA-RNA replication, namely viroids and retrozymes, and provide insights into the potential use of replicating and non-replicating circRNAs in research and biotechnology.

## 2. Non-Replicating Circular RNA in Plants

### 2.1. Back-Splicing and Regulation of Circular RNA Formation

Most known circRNAs encoded by plant genomes are covalently closed products of non-canonical pre-mRNA splicing, referred to as back-splicing. In contrast to normal splicing, which uses an acceptor site located downstream of a donor site, resulting in the excision of an intron located between these two sites, back-splicing uses an acceptor site located upstream of a donor site, resulting in the excision of a covalently closed circular splice product, which may contain exons, with a 3′-5′-phosphodiester bond at the junction site [18,19,20,21]. In addition to nuclear genomic circRNAs, mitochondrial (m-circRNAs) and chloroplast genome-encoded circRNAs (cp-circRNAs) have also been identified; however, the mechanisms of their formation remain unknown [22,23].

For back-splicing to occur, the pre-mRNA regions that will be ligated to form a circular product need to come into close proximity. This can be achieved by annealing inverted repeat sequences flanking the RNA regions excised during splicing [24]. In animals, the inverted repeat sequences surrounding circRNA-forming regions of primary transcripts can vary in size from 30–40 nts to several hundred nucleotides. The degree of complementarity and the length of the repeats influence the efficiency of back-splicing, as well as the timing of its occurrence. In the case of long repeats, cotranscriptional circularization of RNA occurs, whereas in the case of short repeats it occurs after the processing of 3′-end [25,26]. Inverted sequences are often identified as short interspersed elements (SINEs), with Alu elements in primate genomes being a well-documented example [24,27]. Recent studies have reported the presence of inverted repeats flanking circRNAs in some plant primary transcripts. A bioinformatics approach to analysis of maize genomic sequences and RNA-Seq datasets revealed the presence of six types of transposons that were significantly enriched in the flanking regions of circRNAs compared to randomly selected transcripts and frequently located in reverse complementary pairs (RCPs) near back-splicing sites that could facilitate the formation of dsRNA [28]. Furthermore, the presence of RCPs in a plant growth-associated gene was found to positively correlate with the accumulation of a circular back-spliced product and greater plant height, while negatively affecting the formation of a linear canonical splice product, therefore directly demonstrating the functional importance of repeats flanking circRNA [28].

In addition to the cis-acting RNA circularization factors discussed above, there are also trans-acting RNA-binding proteins (RBPs) that interact with flanking intron sequences, bringing them closer together and thereby promoting the transesterification reaction. All such proteins, for which experimental data are available, have been identified in non-plant systems. One such RBP is quaking (QKI), which binds to specific QKI response elements located upstream and downstream of circRNA-forming exons, dimerizes and is responsible for the formation of circRNA during epithelial-to-mesenchymal transition in humans [29,30]. Five *Arabidopsis thaliana* KH-domain-containing proteins have been identified as possessing RNA-binding and dimerization domains that are highly homologous to QKI [31]. However, there are currently no experimental studies of their functions.

CircRNAs encoded by the nuclear genome can be differentially localized in the cell: intron-containing circRNAs, including those derived from linear splicing products, are predominantly nuclear, whereas exonic circRNAs are preferentially cytoplasmic, although some circRNAs are distributed in both compartments [32,33,34]. Numerous studies in animals demonstrated that circRNAs can be secreted from the cell in vesicles into the extracellular space and transported throughout the body [35,36,37,38]. In plants, circRNAs can also be found outside the cell, in the apoplast. However, they predominantly are not present in extracellular vesicles, but rather in the apoplastic fluid, where they interact with proteins that protect them from cleavage and increase their stability. Furthermore, these circRNAs were found to be enriched in N6-methyladenosine (m6A), which may be a consequence of their biogenesis, but may also promote circRNA interaction with RBPs [39].

Despite the absence of a 5′-cap structure and 3′-poly(A) tail, numerous animal circRNAs have been shown to be translatable [40]. In animals, the translation of circRNAs is driven by internal ribosome entry sites (IRESs) and m6A modifications. Recognition of the latter RNA modification can be facilitated by m6A-recognizing RBPs, such as METTL3, YTH domain-containing proteins (e.g., YTHDF1-3), or eukaryotic translation initiation factor 3 subunit h (eIF3h) [11,41,42,43]. Some plant circRNAs are also predicted to be translated. For example, 229 circRNAs with coding potential were found in maize [44], 143 circRNAs with an IRES and 46 circRNAs with a putative translation-promoting m6A modification were discovered in moso bamboo [45], 165 IRES-containing circRNAs were predicted in soybean [46], and ribosome profiling datasets revealed 1569 putatively translatable circRNAs in Arabidopsis [47]. However, experimentally confirmed data on the translation of proteins/peptides from genome-derived plant circRNAs and information on their possible functions are currently lacking. An exception is the plant mitochondria-encoded m-circRNAs, for which a total of 358 m-circRNA-derived polypeptides have been confirmed in maize and Arabidopsis using ribosome profiling data and mass spectrometry (MS) [22]; however, their functions remain to be determined.

### 2.2. Functions of Non-Replicating circRNAs in Plants

The known functions of specific circRNAs correlate with their subcellular localization. For cytoplasmic circRNAs, the main known functions are translation regulation [48], miRNA sponging [49,50], protein retention/decoy, and disruption of protein function [51,52,53,54,55]. The nuclear localized circRNAs can function in transcriptional regulation or splicing [56]. For example, the circular product of the plant SEPALLATA3 gene, which is involved in the splicing regulation of the parental pre-mRNA, is located in the nucleus [57]. The same localization has the human circRNA termed circIPO11, which is known to recruit topoisomerase 1 to the GLI1 transcription factor promoter to activate transcription [58].

A number of recently published studies show differential accumulation of circRNAs at different stages of plant ontogeny and development, which may indicate the global integration of circRNAs into plant signaling systems and responses to external cues [59,60,61,62]. A large number of differentially expressed circRNAs have been found in plants under biotic stress [63,64,65,66,67], and no less number have been associated with the response to abiotic factors [68,69,70,71,72].

Relatively few mechanisms of action are known for plant circRNAs with proven functions. It has been shown that circRNAs derived from exon 6 of the MADS-box-containing gene SEPALLATA3 (SEP3) enhance the generation of the cognate exon-skipped linear alternatively spliced variant. The SEP3 exon 6 circRNA was found to form an RNA:DNA hybrid with the parental gene DNA, a structure termed the R-loop. The circRNA demonstrated a greater ability to form the R-loop than the identical linear product. R-loops are known to form preferentially in GC-rich regions and are presumed to influence a number of processes, including splicing and transcription elongation, which is affected by influencing transcriptional regulatory factors or slowing RNA polymerase II. In addition, they may contribute to the protection of specific loci from DNA methylation and thus from transcriptional silencing. R-loops have also been found to be associated with DNA breaks [73]. In the particular case of SEP3 expression, DNA:circRNA R-loop hybridization may lead to transcriptional pausing, preventing assembly of the complex required for canonical splicing and shifting pre-mRNA processing towards the exon 6-skipped variant (SEP3.3) and the circular form. When overexpressed, the exon 6 circRNA induced changes in AGAMOUS and SEP3 expression levels, particularly a 2.4-fold increase in SEP3.3 expression, and caused morphological changes in the flower, manifested as an increase in the number of petals and a decrease in the number of stamens [57]. R-loop DNA:RNA hybrids are quite common for Arabidopsis chromatin [74], and there are other examples of circRNA-formed R-loops in plants. In maize, three types of circRNAs capable of forming R-loops were produced by back-splicing from transcripts of the centromeric retrotransposon CRM1. In this case, the circRNA formed DNA:RNA hybrids at lower levels than the related linear forms and competed with them for DNA binding. The circRNA-formed R-loop in centromeric DNA promoted the formation of chromatin loops in the centromeric CRM elements, whereas the linear transcript reduced their abundance. In the CRM1 regions, the amount of centromeric histone H3 (CENH3), which is required for maintenance of chromatin function, positively correlated with the amount of circRNA-formed R-loops [75]. It is suggested that the ratio of linear to circular CRM1 transcripts at different stages of the cell cycle is a mechanism for regulating chromatin organization in the centromere region.

Another way in which circRNAs function is through their miRNA sponge activity. This activity is based on the presence of microRNA response elements (MREs) in a number of circRNAs, through which circRNAs can bind complementary miRNAs, reducing their abundance in the cell and relieving inhibition of the targets of these miRNAs [76]. These so-called competing endogenous RNA (ceRNA) or circRNA-miRNA-mRNA networks are widespread in animals, and similar examples are also known for plants. The Arabidopsis ath-circ032768 circRNA, which is derived from the gene of arginine/serine-rich splicing factor 35, has five MREs complementary to miR472. One of the known miR472 targets is the mRNA of RESISTANCE TO PSEUDOMONAS SYRINGAE5 (RPS5), an intracellular receptor of the nucleotide-binding leucine-rich repeat family, members of which are involved in the recognition of pathogen effector proteins and play a role in plant immunity [69,77]. Ath-circ032768 and RPS5 are upregulated after drought stress, whereas miR472 accumulation is downregulated. Overexpression of ath-circ032768 and miR472 sequestration resulted in improved drought tolerance and increased expression of drought stress-responsive genes DERB2A, RD29A, and RD29B [69].

Interactions with various proteins have been demonstrated for some plant circRNAs using co-immunoprecipitation methods. For example, in Arabidopsis, the putative m6A-binding protein GLYCINE-RICH RNA-BINDING PROTEIN 7 (GRP7) and the sRNA-binding protein ARGONAUTE2 (AGO2) were found to interact with extracellular RNAs including circRNAs in the apoplast. It is likely that the interactions are facilitated by m6A enrichment found in extracellular RNAs. Mutations in GRP7 and AGO2 were also found to significantly reduce the amount of extracellular circRNAs. These observations may indicate that GRP7 and AGO2 contribute to the stabilization of circRNAs in the apoplast or to their secretion from the cell [39]. Analysis of AGO immunoprecipitation data also revealed five circRNAs with putative miRNA sponge functions, suggesting that circRNAs may control the post-transcriptional regulation of mRNAs by interacting with AGO proteins [78].

## 3. Viroids

In the early 1970s, circRNAs capable of replication—viroids—were first discovered and characterized [6,7]. Viroids are small (234–467 nt), single-stranded, covalently closed, pathogenic RNA molecules infecting a wide range of agriculturally important host plants [79,80,81]. Unlike viruses that have protein capsids, viroids are naked and presumably incapable of encoding proteins, so they depend on host factors, such as RNA polymerase and processing enzymes, for their replication and pathogenesis [82,83]. *Potato spindle tuber viroid* (PSTVd) was the first viroid discovered and characterized, and since then, 44 viroid species have been described [6,84]. Viroids have been classified into two families, based on their structural characteristics and biological properties. The *Pospiviroidae* family comprises 39 members, with the type species being the PSTVd; viroids of this family adopt a rod-like structure and replicate via an asymmetric rolling-circle (RC) mechanism in the nucleus [84,85]. The *Avsunviroidae* family includes five members, with the type species being the *Avocado sunblotch viroid* (ASBVd), containing hammerhead ribozymes (HHR) in RNA of both polarities that enable self-cleavage during their replication via a symmetric RC mechanism in chloroplasts [84,85].

### 3.1. Main Structural Features of Viroids of the Families Pospiviroidae and Avsunviroidae

**Figure 1:**
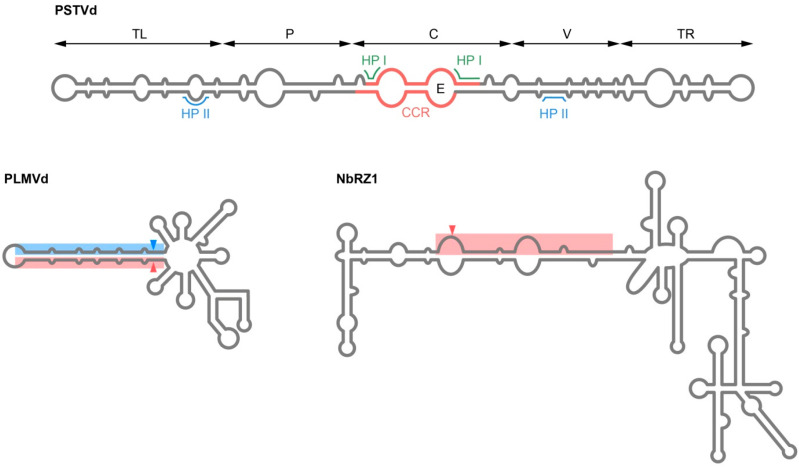
Structural features of viroids and retrozymes. Schematic representation of the rod-like secondary structure of the *Potato spindle tuber viroid* (PSTVd, the family *Pospiviroidae*) genomic RNA shows location of the major PSTVd structural elements. The five structural domains indicated above the structure are as follows: the TL (terminal left), P (pathogenic), C (central), V (variable), and TR (terminal right) domains. A portion of the PSTVd structure shown in red indicates CCR (central conserved region); green and blue lines indicate HPI (Hairpin I) and HPII (Hairpin II), respectively. E indicates E-loop. The multibranched secondary structures of *Peach latent mosaic viroid* (PLMVd, the family *Avsunviroidae*) and *N. benthamiana* retrozyme 1 (NbRZ1) are shown with the positions of regions involved in the formation of hammerhead ribozyme structures in positive and negative polarity strands indicated by red and blue boxes, respectively. The sites of ribozyme self-cleavage are indicated by arrowheads. The NbRZ1 structure shown is a prediction; the structures of PSTVd and PLMVd are supported by experimental evidence.

The family *Pospiviroidae* includes the majority of known viroids; their RNAs adopt similar rod-like structures (Figure 1) formed by two partially complementary halves of the circular viroid RNA, referred to as the “upper” and “lower” strands, in which five domains can be distinguished [81,86]. A central (C) domain of the rod-like structure contains the most conservative motif—central conserved region (CCR), formed by two conserved nucleotide stretches in the upper and lower strands of viroid secondary structure, flanked by an imperfect inverted repeat in the upper strand. The C domain is flanked by a pathogenic (P) domain on the left and a variable (V) domain on the right. In addition, there are two terminal domains, right (TR) and left (TL) [85,86,87,88]. The C domain of many members of the *Pospiviroidae* family contains an internal loop that exhibits homology with the E loop of the eukaryotic 5S rRNA; the structural integrity of this loop is essential for replication and also appears to be involved in host range determination [86,89,90]. Besides these distinctive structural elements characterized for mature viroid RNAs, thermodynamically metastable conformations containing transient structural elements with important biological roles are formed during replication [91]. Such critical for replication metastable structures of Pospiviroids are Hairpin I (HPI), which comprises the upper strand of the CCR along with two flanking inverted repeats, and Hairpin II (HPII), which spans the V and TL domains [91,92].

Members of the family *Avsunviroidae* exhibit a wide range of branched structures within the family. ASBVd, the type species of the family *Avsunviroidae* and the only representative of the genus *Avsunviroid*, is characterized by a high content of A+U and adopts a quasi-rod-like structure, with conserved HHRs in its central domain. The *Eggplant latent viroid* (ELVd) also possesses a quasi-rod-like structure and is classified in the genus *Elaviroid* [85]. A separate genus *Pelamoviroid* includes *Peach latent mosaic viroid* (PLMVd), *Chrysanthemum chlorotic mottle viroid* (CChMVd), and *Apple hammerhead viroid* (AHVd). Despite low sequence identity (ranging from 15 to 38 percent), members of this genus share common characteristics. They possess conserved core nucleotides in HHRs, and HHR sequences face each other in the rod-like catalytic domain fused to a multibranched domain stabilized by interacting loops (“kissing loops”) and pseudoknots [93,94]. In Avsunviroids, catalytically active HHRs, which mediate the self-cleavage of viroid RNA during replication, have been evolutionarily selected to fold into their active conformation only during viroid RNA transcription and are not active in the most stable conformation of mature viroids [88,95] (Figure 1).

### 3.2. Viroid Replication

Viroids undergo RNA-RNA replication, without DNA intermediates, via an RC mechanism, with some differences between the two families. The most common and frequently encountered form of viroid RNA is conventionally considered to have positive polarity, while its complementary strand has negative polarity. In the first replication step, longer-than-unit oligomeric linear molecules are synthesized by a host polymerase on a template of circular genomic single-stranded (+) RNA [96,97]. In the next step, members of the *Pospiviroidae* family use an asymmetric RC mechanism in which (−) sense oligomers are first used as templates for the synthesis of (+) oligomers, which are in turn processed to monomers and circularized [96]. Members of the *Avsunviroidae* family follow a symmetric RC mechanism in which linear (−) sense oligomers are first processed by HHRs and circularized into monomeric (−) RNAs, serving as templates for the synthesis of linear oligomeric (+) RNAs, which in turn are processed into monomers and circularized to form mature viroid (+) RNAs.

Since viroids are currently not shown to encode proteins, they must rely on RNA structures to engage host mechanisms for successful infection. The first step following entry into the plant cell, as a result of the mechanical injury, for members of the *Pospiviroidae* family, is their active transport into the nucleus, which is independent of microtubules and actin filaments, but appears to depend on interactions with other host factors [98]. In the case of PSTVd, the cellular BROMODOMAIN-CONTAINIG PROTEIN 1 (VirP1), which contains a nuclear localization signal, has been shown to specifically bind to the C-loop in the TR domain and regulate PSTVd replication [99,100,101]. This protein has been identified as a promising candidate for the role of a cellular factor that directs viroid transport to the nucleus [102,103]. Considering that VirP1 is able to mediate the nuclear import of the *Cucumber mosaic virus* satellite RNA, which also contains a C-loop, it has been suggested that this protein may effectively bind to the C-loop conserved among nuclear-replicating viroids and presumably direct nuclear import via the importin alpha4-based pathway [104]. However, as PSTVd has been described to have additional structures in the upper strand of CCR and HPI involved in nuclear transport, it seems likely that nuclear transport is supported by multiple pathways employing additional cellular factors and/or viroid signals [85,104,105].

Upon reaching the nucleoplasm, Pospiviroids recruit DNA-dependent RNA polymerase II (Pol II) and reconfigure it to accept RNA templates using a plant-specific splicing isoform of transcription factor IIIA (TFIIIA-7ZF). This reconfiguration results in a reorganization of the Pol II complex, leading to a reduction in the number of components compared to the canonical 12- or 10-subunit Pol II core on DNA templates [84,106]. Interestingly, the reorganized complex lacks the Rpb9 subunit, which is critical for fidelity. This could explain the increased mutation rate observed in viroids as compared to in the cellular transcripts [97,106].

Pol II-mediated transcription results in the formation of linear (−) sense oligomers, which serve as templates for the synthesis of linear (+) sense oligomers. Although synthesis of the (+) strand on the template of (−) strand is also thought to be catalyzed by Pol II, this has not yet been clearly demonstrated [107]. The oligomeric (−) strands of PSTVd accumulate only in the nucleoplasm, whereas (+) strands are distributed between the nucleoplasm and the nucleolus. This suggests that transcription of both strands occurs in the nucleoplasm, and the (+) strands are then transferred to and processed in the nucleolus [108]. Therefore, a mechanism is thought to exist that can distinguish between strands of the two polarities. In the nucleolus, (+) sense oligomers are cleaved into monomers. This process involves the formation of a metastable conformation, in which a site in the upper strand of the CCR, together with a short flanking inverted repeat, forms an HPI structure including the terminal capping tetraloop with conserved CG central residues. Two consecutive viroid units in the oligomeric intermediates interact via “kissing loops” between tetraloops of HPI motifs, thereby initiating intramolecular dimerization. This quasi double-stranded structure is recognized by an RNase that cleaves the interstrand duplex, releasing a monomer [109]. The cleavage site is located between nucleotides G96 and G97 in the upper strand of the PSTVd CCR or between two G’s at similar sites in other viroids [109]. Based on a study in which (+) RNA monomers resulting from cleavage of a dimeric transcript transgenically expressed in Arabidopsis exhibited features such as 5′-phosphomonoester and 3′-hydroxyl termini corresponding to RNase III cleavage products, RNase III is considered the most likely enzyme to cleave (+) sense oligomers into monomers [110], but there is currently no direct in vitro or in vivo evidence that this enzyme actually cleaves viroid RNAs. The cleavage is accompanied by the formation of a multibranched metastable hairpin (distinct from Hairpin I), capped by a GAAA tetraloop conserved in members of the genus *Pospiviroid*, which subsequently switches to a stable conformation—loop E [111]. Loop E facilitates ligation by helping to position the termini in close proximity and in the correct orientation for DNA ligase I-mediated ligation, resulting in the formation of mature viroid RNA [109,112]. Since loop E is not conserved in *Pospiviroidae*, it remains to be determined whether DNA ligase I relies on loop E or on a more general structure in the CCR.

Viroids of the *Avsunviroidae* family must be transported to the chloroplast for replication, which is associated with the thylakoid membrane [113]. However, the exact mechanism of this transport remains to be elucidated. Interestingly, some data suggest that after entering the cytoplasm, members of the *Avsunviroidae* family may be transported first to the nucleus and then to the chloroplasts. In particular, an artificial mRNA, in which the ELVd sequence is placed upstream of the GFP-coding sequence acting as a 5′-untranslated region, can be specifically transported from the nucleus to the chloroplasts [114,115,116]. A structural domain of ELVd responsible for this transport overlaps with the region responsible for the nuclear localization of ELVd. Thus, it is likely that a single RNA sequence and/or structure in ELVd mediates both the nuclear and subsequent chloroplast localization of ELVd [116]. The ELVd mutant carrying a deletion in this region is unable to infect its natural host, although it undergoes cleavage and ligation in vivo in chloroplasts [117]. Taken together, the limited data available suggest that the intracellular trafficking of Avsunviroids appears to be a complex multi-step process, but the host factors involved in this process are unknown.

ASBVd replication has been shown to be insensitive to α-amanitin and tagetitoxin, which are known to inhibit the plastid-encoded polymerase (PEP), but not the nuclear-encoded polymerase (NEP), making NEP the most likely candidate for mediating replication of ASBVd and possibly other viroids of this family; however, the involvement of another chloroplast RNA polymerase that is resistant to tagetiotoxin cannot be excluded [118]. NEP is a nuclear-encoded polymerase that is transported to the chloroplast and functions there during chloroplast development; therefore, it could also be a potential factor involved in the transport of Avsunviroids from the nucleus to the chloroplast [119]. It is noteworthy that the mutation rate of viroids of the family *Avsunviroidae* is the highest among all biological entities. This may be a consequence of errors introduced by NEP during transcription of non-physiological RNA templates and the absence of the polymerase proofreading activity [120]. However, the mechanisms of NEP recruitment and whether this is a site-specific process remain unclear, as transcription start sites differ among family members.

The replication of (+) sense Avsunviroid genome results in (−) sense oligomers, which are co-transcriptionally processed to monomers by HHR-mediated self-cleavage. HHRs catalyze a sequence-specific intramolecular transesterification reaction starting with a nucleophilic attack of the 2′-oxygen on the adjacent 3′-phosphate, resulting in the cleavage of the phosphodiester bond and the formation of two RNA products with a 5′-hydroxyl and a 2′,3′-cyclic phosphate, respectively [121]. The active structure of HHR is represented by a catalytic core consisting of 15 highly conserved nucleotides flanked by three double-stranded helixes with non-strict sequence requirements (I to III), where helixes I and II are capped by a short loop and interact with each other through non-canonical interactions between nucleotides in the helixes. These interactions, which apparently require the presence of divalent metal ions such as Mg^2+^, stabilize the active conformation of HHRs and are essential for ribozyme activity [121,122,123,124]. X-ray crystallography data indicated that HHRs adopt a Y-shaped configuration with helix III positioned almost co-linearly with helix II and helix I, forming an angle due to a classical uridine turn structure [121,122,125]. HHRs are classified as type I, II, or III, depending on whether the 5′ and 3′ termini are located within helix I, II, or III, respectively. Viroids and viroid-like RNAs are characterized by HHR type III motifs [125].

The monomers are efficiently circularized by a chloroplast isoform of the host tRNA ligase, as demonstrated for the (+) and (−) strands for at least four family members in vitro and for ELVd in vivo [112]. The enzyme is shown to specifically recognize the 5′-hydroxyl and 2′,3′-cyclic phosphodiester termini of RNA formed by HHRs and to efficiently circularize monomeric (+) sense ELVd RNA. The precise mechanism, by which the chloroplast isoform of the tRNA ligase recognizes and interacts with the viroid, is not fully understood. However, one study points to the importance of a quasi-rod-like structure in the central part of ELVd, which contains the ligation site in an internal loop, in this process; another study suggests that the HHR also contributes to this recognition in addition to its primary function [85,117,126].

### 3.3. Viroid Intercellular and Long-Distance Transport

To infect the whole plant, viroids must first be transported from infected to healthy cells through plasmodesmata and then into the phloem for long-distance transport. Structural features of viroid RNA are believed to be of critical importance for interaction with host factors that enable viroid transport in plants. Using PSTVd as a model, such features have been shown to represent local three-dimensional (3D) structural motifs, which are formed in imperfect RNA duplexes by interacting RNA nucleotides involved in the formation of both canonical Watson−Crick base pairs and other types of bonds; such 3D motifs provide structurally unique surfaces for interaction with proteins [127]. Individual 3D motifs appear to specifically determine PSTVd transport across boundaries between different cell types in plant tissues, suggesting that different PSTVd-binding proteins are involved in different steps of transport in plants. In particular, mutational analyses have shown that the right terminal loop (loop 27) of PSTVd RNA is critical for its transport from the epidermis to the palisade mesophyll cells [128]; loops 6 and 19 facilitate transport from the palisade mesophyll to the spongy mesophyll [129,130]; and a bipartite motif comprising U201 in the TR domain and U309/U47/A313 in the P domain is required for transport from the bundle sheath to the mesophyll [131]. To date, the host factors that interact with the 3D motifs to allow viroid to move across specific cell boundaries are poorly investigated. The chaperone-type cucumber PHLOEM PROTEIN 2 (CsPP2), which is homologous to the *Cucurbita maxima* PP2 known for its ability to increase the conductivity of plasmodesmata channels and to allow its own transport from the companion cells to the sieve tubes, has been shown to form a complex with *Hop stunt viroid* (HSVd) in vitro and to promote viroid transport across the graft union [132,133]. Furthermore, an interaction between CsPP2 and the *Apple scar skin viroid* (ASSVd) has been demonstrated in vivo [134]. These findings suggest that PP2 proteins may have the ability to bind viroids and promote their phloem transport. Two phloem proteins capable of binding the viroids and transporting across graft unions have also been described for the chloroplast viroid ASBVd [135]. In addition, a possible role of the Nt-4/1 protein in the long-distance phloem transport of PSTVd has been reported [136]. These findings fit well with the concept that viroid RNA specifically interacts with plant protein factors directly involved in its transport. In addition, an indirect mechanism for regulating viroid trafficking in plants has been described. Small RNAs produced from virulence modulating loops 7 and 8 of PSTVd appear to regulate transport by their ability to silence the callose synthases CalS11 and CalS12, which regulate transport through plasmodesmata [137].

### 3.4. Protein-Coding Capacity of Viroid RNA

Since the discovery of viroids, much effort has been devoted to identifying their translational capacity, which could be more easily linked to the dramatic effects that these small non-coding circRNAs have on plant biology. Interestingly, a wealth of evidence points to their influence on and interaction with the cellular translation machinery. For example, CEVd infection has been shown to alter the expression of proteins such as the ribosomal proteins S3, S5, and L10, as well as the eukaryotic translation factors eEF2, eIF5A, and eEF1A, with the latter shown to interact directly with CEVd [138]. eEF1A is also detected in PLMVd-binding protein assays, and such a PLMVd-eEF1A complex has even been isolated from leaves and further characterized [139]. CEVd infection appears to affect ribosome function and even induce ribosomal stress. The viroid is found in polysomal fractions and impairs the maturation of 18S rRNA and consequently the 40S ribosomal subunit [140,141]. Similar effects on ribosome biogenesis and ribosomal stress induction have recently been shown for *Tomato chlorotic dwarf viroid* (TCDVd) and PSTVd [142].

Several recent studies have revisited the non-coding nature of viroids and attempted to detect their translation products. The results showed that both HSVd and ELVd viroids not only associate with the cell translation machinery, but also possess conserved open reading frames (ORFs) that are potentially capable of encoding peptides [16]. The corresponding ORFs were fused to the GFP gene and transiently expressed in *Nicotiana benthamiana* plants. The results demonstrated that the peptide of the nuclear-replicating viroid was localized to the nucleus, whereas the peptide of the chloroplast-replicating viroid was localized to the chloroplasts, indicating their potential functional role. In addition, several mutant variants of these peptides with prematurely introduced stop codons reduced the levels of HSVd and ELVd RNA accumulation. However, attempts to detect the predicted peptides by liquid chromatography−MS (LC−MS) in total protein extracts from infected tissues were unsuccessful [16]. In another recent study, Katsarou and co-authors performed a comprehensive bioinformatics analysis of 30 different *Pospiviroidae* species and found that all contained small ORFs with average predicted peptide sizes ranging from 3 to 15 kDa. The circular PSTVd genome was shown to localize to ribosomes in both *N. benthamiana* and tomato. However, no viroid-specific peptides were found to be expressed. Therefore, it has been suggested that viroids use ribosomes for functions other than translation, such as protection of viroid RNA from endonucleases by binding to ribosomes or microtubule-mediated transport of ribosome-bound viroid [143].

### 3.5. Molecular Mechanisms of Viroid Pathogenesis

Viroids cause severe symptoms in infected plants, including stunting, chlorosis, and deformation of leaves, fruits, and flowers. A simple explanation for the phenotypic effects of viroid infection may be the direct recruitment of host cellular factors to the viroid life cycle. However, recent evidence suggests that the severe symptoms of viroid infection can be attributed, at least in part, to the defense response to viroid infection. In fact, viroid RNA, which has an extensive secondary structure, is targeted by the RNA-silencing machinery. Viroid-derived small RNAs (vd-sRNAs) of various sizes have been detected in plant tissues infected with both nuclear- and chloroplast-replicating viroids [91]; such vd-sRNAs could potentially act as miRNAs, directing specific cleavage of essential cellular mRNAs, thereby reducing the expression of corresponding proteins and causing severe symptoms. This mechanism was first demonstrated for two PLMVd strains, PLMVd-PC and PLMVd-PYM, which induce different symptoms in infected plants and differ in the sequences of their pathogenicity determinants; they give rise to different specific vd-sRNAs that mediate the cleavage of two unrelated mRNAs and consequently disrupt the expression of different proteins. Specifically, PLMVd-PC directs the degradation of the chloroplast heat shock protein HSP90 mRNA, whereas PLMVd-PYM targets the thylakoid translocase subunit cpSecA mRNA [91,144,145]. Since then, a large number of mRNA targets for different viroids have been identified. Notably, the majority of vd-sRNAs are generated from regions previously described as pathogenicity determinants that may be due to the secondary structures of these regions being more susceptible to processing by the RNAi machinery [85]. The identified targets include a wide range of mRNAs that encode proteins with diverse functional roles, including the following: (a) transcription factor TCP23, which is involved in plant development and hormone regulation; (b) callose synthases Cal-S11 and Cal-S12; (c) chloride channel protein CLC-b-like; (d) ribosomal protein S3a; (e) flowering-related protein 3 (FRIGIDA-like protein 3); (f) host receptor serine-threonine kinase (RSTK); and (g) other proteins involved in plant defense and development [137,146,147,148,149].

In addition, viroid-induced changes in plant gene expression at the transcriptional level may contribute to the development of viroid infection symptoms. A correlation between viroid infection and methylation changes in the plant genome has been found. Specifically, HSVd infection in *Cucumis sativus* and *N. benthamiana* can induce changes in the DNA methylation pattern of host rRNA-encoding genes. During HSVd infection, an increase in the transcription of rRNA precursors is observed, which correlates with a decrease in DNA methylation in their promoter region [150]. Moreover, demethylation appears to be associated with the suppression of histone deacetylase 6 (HDA6) activity caused by HSVd binding to this protein. Interestingly, a previous study demonstrated that in Arabidopsis *hda6* mutants, Pol II was able to transcribe non-canonical rDNA templates that are normally transcribed by RNA Pol I [151]. Therefore, it was proposed that HSVd could bind and functionally inactivate HDA6 to promote the misrecognition of HSVd RNA as a template by RNA Pol II, thereby facilitating viroid replication [152].

Thus, the pathogenesis of viroid infection and associated symptoms appears to be a complex and multifaceted process. As a result, viroid infections affect the expression of multiple genes associated with different plant systems, including photosynthesis, plant hormone signaling, translation, protein metabolism, RNA processing and binding, as well as genes involved in plant innate immune responses such as mitogen-activated protein kinase, pathogen-related genes, and others [85].

### 3.6. Viroid-like RNAs

Speaking of viroids, it is worth mentioning such a class of subviral agents as small circular satellite RNAs (sc-satRNAs), which are also referred to as viroid-like RNAs due to common features with viroids, including a small (220–257 nt) single-stranded genome, accumulation in the infected host in the form of covalently closed circRNAs, replication by a RC mechanism with only RNA intermediates, self-cleavage by HHRs, and an inability to encode proteins (with one exception) [153]. The major biological difference between viroids and sc-satRNAs is that the life cycle and replication of sc-satRNAs depend not only on plant factors, but also on a helper virus. This virus provides the necessary RNA-dependent RNA polymerase that replicates the satellite RNAs, as well as the viral capsid protein for their encapsidation [153]. Interestingly, sc-satRNAs can contain ribozymes, and in some cases, each strand contains its own type of ribozyme [154].

It is also worth noting that there is a distinct and unique case of viroid-like RNA, the so-called retroviroid, which is a Carnation small viroid-like (CarSV) RNA, 275 nucleotides long, which also occurs in a DNA form [155]. The RNA form of CarSV RNA shares structural features with viroids and viroid-like satellite RNAs. It exists as a covalently closed circular molecule, replicates autonomously and has HHRs in strands of both polarities that allow self-cleavage. The DNA form of CarSV is organized as a series of head-to-tail multimers that are part of extrachromosomal elements in which the CarSV DNA sequences are surrounded by short sequences derived from a plant pararetrovirus, *Carnation etched ring virus* (CERV) [155]. One hypothesis for the origin of such a unique retroviroid element is that during coinfection of an ancient ancestor of CarSV RNA (which later lost virulence) and CERV, CarSV RNA may have been reverse transcribed by the CERV-encoded enzyme to produce the CarSV DNA. Based on the analysis of the junction regions, it can be speculated that this event may have resulted from reverse transcriptase (RT) template switching in the 4–6 nucleotide regions of homology. It has been demonstrated that CarSV RNA cannot be transmitted horizontally and is not capable of causing infection by itself, suggesting that the transcripts are derived from the CarSV DNA form [156].

## 4. Retrozymes

### 4.1. Structure and Origin of Retrozymes

Another type of endogenous circRNA, capable of autonomous replication in plants, is the RNA of retrozymes (retrotransposons with hammerhead ribozymes). These retroelements, first identified in plant genomes, have sizes ranging from 1 to 1.5 kb and a characteristic structural organization. The complete plant retrozyme is delimited by two target site duplications (TSDs) and consists of two direct long terminal repeats (LTRs) with embedded type III HHRs and a central region containing the primer-binding site (PBS) (tRNA-Met binding site) and a poly-purine tract (PPT), both necessary for DNA synthesis during the mobilization of LTR-retrotransposons [12,157]. It should be noted that such complete elements have only been identified in dicotyledons, although the presence of putative retrozyme sequences has also been detected in primitive land plants and algae [158]. It is suggested that a selection pressure acts at the level of maintenance of a specific retrozyme RNA structure and stability, since retrozyme elements in different plants show no sequence identity, except for the short PBS, PPT, and HHR motifs that function at the RNA level [12]. Retrozymes are non-autonomous retroelements, and like other similar retroelements identified in plant genomes such as TRIMs (terminal-repeat retrotransposons in miniature) and SMARTs (small LTR-retrotransposons), their mobilization depends on the reverse transcriptase (RT) encoded by autonomous retrotransposons, most likely those of the Ty3-gypsy family [159,160]. Plant retrozyme transcripts contain two LTRs with embedded type III HHRs. After transcription, ribozymes first generate a linear RNA intermediate with a 5′-hydroxyl and a 2′,3′-cyclic phosphate ends, which is then ligated by tRNA ligase to form a circular retrozyme RNA [12]. Subsequently, the circRNA of retrozymes is replicated by an RC mechanism. Replication products can be further used as templates for reverse transcription (cDNA synthesis) with subsequent integration into a new genomic locus [12]. For cDNA synthesis and retrotransposition to a new genomic locus, retrozymes require activity of integrase (INT) and RT, which are encoded by autonomous retrotransposons. It is important to note that cDNA produced by retrotranscription can have different lengths, depending on the processivity of RT. As a result, single copies of retrozyme elements and tandem copies of several retrozyme genome units, as well as single LTRs, are found in plant genomes [12,13,14].

Initially, retrozymes were identified only in plant genomes, but later, similar elements were discovered in vertebrate and invertebrate genomes. Metazoan retrozymes are non-autonomous, non-LTR retroelements that are approximately 170–400 nucleotides in length, contain type I HHR and are capable of circularization [161]. As in the case of LTR-containing plant retrozymes, the non-LTR metazoan retrozymes encode no RT necessary for their own mobilization. Therefore, they can only be mobilized by some other active members of the families of non-LTR retrotransposons, such as LINEs or Penelope-like retroelements (PLEs) [162,163,164].

Circular and linear retrozyme RNA intermediates have been shown to accumulate at a high level in different tissues of analyzed plants [12,13]. However, it is not clear whether these RNA molecules result from active transcription of the corresponding genomic retrozyme loci. It was suggested that the linear RNA products detected by Northern blotting could be the result of primary transcription, while the circRNAs of retrozymes could be the products of replication by the RC mechanism with subsequent processing of the formed product by the activity of ribozymes in the retrozyme RNA and circularization [12]. Later, it was shown that the LTR of the *N. benthamiana* retrozyme-1 (NbRZ1), containing a promoter, is hypermethylated, and an active transcription is unlikely to occur at this locus [13]. However, it should be noted that activation of retroelements without changing the level of promoter methylation is possible, as was shown for *A. thaliana* LTR-retrotransposon ONSEN [165]. The existence of another level of transcription regulation associated with the functions of Methyl-CpG-Binding-Domain (MBD) proteins was identified [166]. MBD2 interacts with genomic regions containing high levels of CG methylation, and MBD2 silencing in plants was found to lead to the activation of mobile elements without changing the methylation level of the corresponding genomic loci [166]. While the question of the retrozyme loci transcription remains disputable, the replication of retrozyme RNA in plants was demonstrated using a cloned NbRZ1 sequence containing the introduced marker sequence [13]. Interestingly, the active replication of retrozyme RNA was found to lead to the accumulation of sequence changes. In addition, retrozyme RNA was shown to be capable of systemic transport similar to viroids [13]. Therefore, it can be assumed that the observed abundance of retrozyme RNA in different organs and tissues of the analyzed plants may not be the result of primary transcription, but of RNA-RNA replication by the RC mechanism, as well as the systemic spread of the progeny retrozyme molecules [13]. This suggestion is consistent with the concept that retroelements are transcribed only at certain stages of plant development or under stress conditions [167,168].

The genomic HHRs and retrozymes present in modern angiosperms and metazoans could be evolutionarily derived from Penelope-like retroelements (PLEs) [14], which are present in primitive *Selaginella* plants as well as in vertebrate and invertebrate genomes and are believed to predate most eukaryotic retrotransposons [163,164]. Genomic PLEs are delimited by two direct repeats (PLTRs), which contain spliceosomal introns and flank a single ORF with RT and endonuclease domains [169,170]. Interestingly, metazoan retrozymes and PLEs share some common structural features, such as the presence of type I HHRs [171], their existence as tandem copies, and their coexistence in all metazoans analyzed [161,169]. It can be proposed that the retrozyme elements identified in the genomes of modern plants and animals could be considered as simple genomic parasites that arose as a result of the genome reduction of PLEs.

Viroids and some viroid-like agents have been proposed as relics of the prebiotic RNA world because of their simplicity, ability to autonomous replication, and the availability of ribozymes [172,173]. Alternatively, according to the Diener’s early hypothesis, such agents might have arisen through genome escape, i.e., a circRNA encoded by the cell genome could have acquired the ability to replicate autonomously while the corresponding genomic locus was subsequently lost in evolution, which seems to be the most likely scenario for the emergence of viroids [6]. In line with this view, the hypothesis of the evolutionary origin of HHR-containing viroids from retrozymes appears to combine elements of escape and reduction scenarios, and it is likely that these agents could have originated from cellular genomes [154,174]. In recent years, evidence has emerged that suggests the likelihood of such a scenario. There are similarities between retrozymes and Avsunviroids in their biology. Both replicate via the RC mechanism, contain the same type III HHR motifs and are probably ligated by tRNA-ligases [12,112]. Furthermore, nucleotide sequence substitutions are rapidly accumulated during replication and persistence both viroids and retrozyme RNAs in plants [13,120,175]. Moreover, retrozyme RNA, as noted above, is capable of long-distance transport in plants like viroids [13]. The RNA sequences of retrozymes are predicted to fold into stable, branched structures [12] similar to those of Avsunviroids, and RNAs of most retrozymes and viroid-like sequences have both high G-C content (55%), with guanine as the most abundant nucleotide (30%) and adenine and uracil at about 22% each [14]. To summarize, the retrozyme escape hypothesis seems to be the most likely scenario for the origin of HHR-containing viroids [15]. Alternatively, retrozymes could either be HHR viroid-related elements integrated into the genome [93,176], or they could be similar to viroids as a result of convergence.

### 4.2. Protein-Coding Capacity of Retrozyme

In recent years, there has been a remarkable increase in the characterization of novel types of circRNAs, as well as in the understanding of their mechanisms of action and functions in development [8,177] and in response to various stresses [178]. It has been demonstrated that plant circRNAs have the potential to serve as templates for protein and peptide synthesis [47]. However, the translation mechanism that can be used for such RNAs must be cap-independent, since the template is covalently closed [8]. It is suggested that such templates can be translated through the ability of m6A to recruit translation factors without the involvement of cap and poly(A) under heat shock conditions [179]. Further studies demonstrated that m6A-dependent translation of circRNAs is facilitated by the recruitment of the m6A reader protein YTHDF3, along with translation initiation factors eIF4G2 and eIF3A [11]. An alternative mechanism could be IRES- or IRES-like element-dependent translation [180]. This suggestion is supported by observations that IRESes are predicted in circRNAs with potential coding capacity in plants and animals [46,181] and provide high levels of protein production in recombinant circRNAs [182]. Furthermore, it has been demonstrated that structured RNA of HHRs can serve as an IRES-like element, facilitating cap-independent translation; in addition, an analysis of the RNA structural comparison between HHRs and the core domain of picornavirus IRES elements has revealed some structural similarity [183].

According to ribosome profiling (Ribo-Seq) datasets, numerous plant circRNAs are associated with ribosomes and have protein- or peptide-coding capacity, but there is currently no experimental evidence that these RNAs are translated [8,47]. There are several possible reasons for the lack of experimental data on the translation of plant circRNAs. First, it is possible that cap-independent translation mechanisms are so inefficient in the case of circRNAs, operating mainly under stress conditions, that even if such products are translated, they are too scarce to be directly detected by MS [180,184]. Second, the translation products of circRNAs may be mostly aberrant and are very rapidly removed from the cellular protein pool, forming a “hidden proteome” [180,185]. Clearly, further studies are needed in this field.

Given the distribution of retrozyme among different plant taxa, their relatively high copy number, and the fact that retrozyme RNAs are persistent and actively replicate, it seems reasonable to hypothesize that retrozyme RNAs may have acquired a specialized function. One such function of retrozyme circRNAs may be the ability to encode functional proteins, as the RNAs of *N. benthamiana* retrozyme elements contain an ORF (our unpublished data); however, there are no experimental data on the translation of this ORF in plants. Similarly, there is no definitive information on the translation of small ORFs found in viroids (see above).

In this context, it is interesting to mention the protein-coding capacity of some circRNAs identified among a large variety of viroid-like and viral circRNAs found in different transcriptomes [177,186]. One of these circRNAs, belonging to the unclassified genus *Ambivirus*, contains HHR and hairpin (HPRz) ribozymes and two ORFs, one of which encodes RNA-dependent RNA polymerase (RdRp) and is closely related to those found in *Orthornavirae* [187]. Although the mechanism of ambiviral circRNA translation has not been studied, they have been shown to be able to replicate in fungi that is likely associated with the RdRp activity [188]. It is worth mentioning that among the identified circRNAs containing self-cleaving ribozymes, some mitovirus-like RNAs have been found [177]. All identified mitovirus-like circRNAs contain an ORF with sequence similarity to the RdRp of mitoviruses [188]. Further work is therefore required to demonstrate that mitovirus-like circular RNA can serve as a template for RdRp synthesis. Currently, to the best of our knowledge, the only example that convincingly demonstrates the ability of circular viroid-like RNAs to be translated in vitro and in vivo is the virusoid associated with *Rice yellow mottle virus* (genus *Sobemovirus*), which encodes a unique 16 kDa protein that has no sequence similarity to any known protein and is capable of RNA-binding [17]. It can be concluded that viroid and viroid-like RNAs including retrozyme RNA have the potential to encode functional proteins; however, the current experimental data are limited, and a definitive conclusion has yet to be reached.

### 4.3. Probable Functions of Retrozyme in Systemic Signaling, Stress Response, and Plant Development

Another function of retrozyme circRNAs may be associated with systemic signaling, as it has been shown that retrozyme RNA is capable of long-distance transport in *N. benthamiana* plants [13]; however, the potential target of such regulation is unknown. In line with this observation, *A. thaliana* apoplastic fluid has been shown to contain RNA-binding proteins and circRNAs, indicating that these RNAs are capable of transport through the plant body; as in the case of retrozyme circRNAs, the functions and signals that direct the long-distance transport of such RNAs have not yet been elucidated [39]. To date, two types of signals in RNA molecules that direct their long-distance transport have been identified. One type of signals is represented by specific sequences, such as the polypyrimidine tract [189], while another is represented by elements of the secondary/tertiary structure forming structurally unique 3D motifs, as shown in for PSTVd model (see above). Signals of both types are believed to be specifically bound by proteins that direct the selective long-distance transport of signal-containing RNAs. In the case of the polypyrimidine tract, which is required for long-distance transport of a number of mRNAs [190,191], this role is played by polypyrimidine-binding proteins. For example, in potato plants, it has been shown that leaf-derived StBEL5 transcripts move through the phloem to stolon meristems in response to changes in day length [192] and that the RNA-binding proteins StPTB1 (*Solanum tuberosum* POLYPYRIMIDINE TRACT-BINDING1) and StPTB6 mediate this movement [193].

Among the 3D motif-based signals of systemic transport, such as those in tRNA-like structures in genomic RNAs of a number of plant viruses [194,195], PSTVd RNA [196], cellular tRNAs and tRNA sequences within mRNA untranslated regions [197,198], as well as in some pri-miRNAs [195,199], the best studied is the signal in PSTVd RNA. A signal that directs long-distance RNA transport of retrozymes remains to be elucidated. However, the similarity of retrozymes to viroids suggests that the signal may be an element(s) of secondary-tertiary structure, probably similar to those identified for PSTVd.

The promoters of a number of plant retroelements have been shown to contain regulatory elements that activate transcription under stress conditions and are likely to bind specific transcription factors (TFs). *AtCOPIA4* is located in close proximity to a cluster of genes-encoding proteins involved in the plant response to pathogens, known as resistance (R) genes [200]. In particular, *AtCOPIA4* is adjacent to the RPP4 gene which confers resistance to the downy mildew oomycete *Hyaloperonospora parasitica,* and mutations in *AtCOPIA4* significantly reduce plant resistance to this pathogen [200]. It seems reasonable to propose that this may be because the *AtCOPIA4* promoter contained in the LTR comprises binding sites for WRKY TFs, which are known to orchestrate transcriptional reprogramming during pathogen-associated molecular pattern (PAMP)-triggered immunity (PTI) [201]. The insertion of the LTR-retrotransposon *ONSEN*, which has been demonstrated to be reactivated upon heat stress [202], into a new genomic locus confers heat-responsive properties to genes in the new locus [203]. It can be suggested that this is also a consequence of the activation of promoter contained in the LTR, as previously observed in the case of the two LTR-retrotransposons, PHRE1 and PHRE2 of *Phyllostachys edulis*, whose 5′ LTRs contain promoters that are activated during the response to heat stress and interact with heat-dependent TFs [204]. In the case of retrozymes, the NbRZ1 promoter has been shown to be hypermethylated, indicating that the retrozyme promoter is barely constitutive. It can be proposed that it is activated in response to some stress or at a certain stage of plant development and that this activation, probably transient, results in generation of retrozyme transcripts that subsequently persists in plants through its ability for replication as well as intercellular and long-distance transport [13].

It can be speculated that a possible regulatory function of retrozyme RNAs in plants may be associated with their potential to act as miRNA sponges under stress conditions in order to maintain normal plant growth and development. Indeed, circRNAs have been shown directly in animal models and more recently in plants to act as miRNA sponges, regulating the level of accumulation of different mRNAs by modulating the amount of complementary miRNAs through the presence of miRNA-binding sites [8,50,69]. Furthermore, evidence indicates that linear RNAs of certain plant retroelements may exhibit similar miRNA-sponging function. The *MIKKI* retrotransposon has been shown to be actively transcribed in rice roots and to have a significant impact on root system development. The *MIKKI* RNA contains multiple introns and has low coding potential, and splicing generates a binding site for miR171 at the exon−exon junction. Active transcription of this element in roots results in depletion of the miR171 pool and, as a consequence, maintenance of the required level of accumulation of SCARECROW-like mRNAs. Thus, the linear transcript of this retrotransposon has been shown to act as a miRNA sponge that is essential for maintaining of proper root development [205].

On the other hand, stem-loop structures and double-stranded replicative intermediates of active retroelements may be sources of small RNAs that regulate some physiological processes in plants. For example, it has been shown that the *ATHILA6* retroelements, activated under stress conditions, can act as a source of siRNA854 specific to the mRNA of the UBP1b protein, which is a component of stress granules associated with translation inhibition in the cell [206,207]. The formation of these granules in plant cells is a defense mechanism in response to transposon activation and virus infection aimed at inhibiting them [206,208]. In rice, plant resistance to infection by *Xanthomonas oryzae* pv. *oryzae* has been shown to be mediated by the WRKY45 transcription factor. Two alleles of this gene have been identified, exhibiting opposite effects. Expression of WRKY45-2 conferred resistance of rice plants to this pathogen, while expression of WRKY45-1 rendered the plants susceptible. Further study revealed that this effect is associated with the presence of the *WANDERERER* retroelement in the WRKY45-1 gene intron. The presence of this element resulted in generation of 24 nt siRNA815 and sRNA-directed DNA methylation of the genomic locus of the ST1 gene, an important component of WRKY45-mediated resistance [209,210]. It is worth noting that the accumulation of small 21 and 24 nt RNAs derived from retrozyme RNAs in plants has also been demonstrated [13]. These small RNAs can potentially modulate the accumulation level of corresponding mRNAs, but the targets of such regulation have yet to be identified.

**Figure 2:**
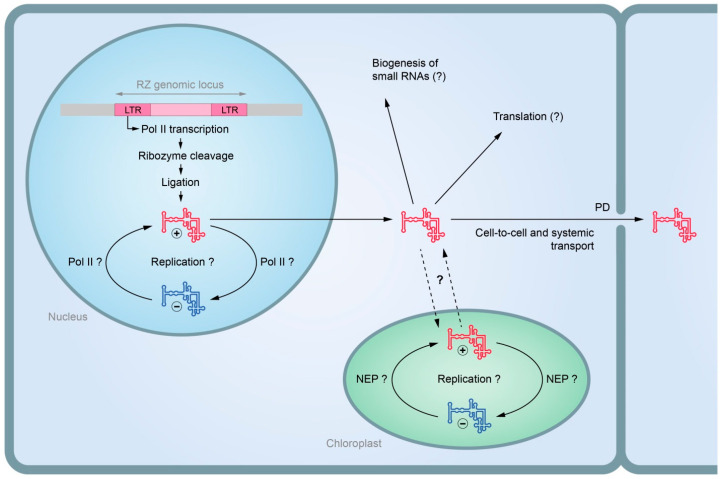
Proposed model for the retrozyme life cycle in plants. In the nucleus, activation of the retrozyme promoter located in the retrozyme (RZ) LTR and transcription of the RZ genomic locus results in the retrozyme transcript. The RZ transcript is cleaved by ribozymes located in the LTRs; the resulting RNA has a 5′-hydroxyl and a 2′,3′-cyclic phosphate end, which are ligated to give the retrozyme circular form. Since the site of retrozyme RNA replication is unknown, it has been proposed to occur either in the nucleus or, in analogy to Avsunviroids, in the chloroplast where the retrozyme RNA may be transported after exit from the nucleus to the cytoplasm, as indicated. In the nucleus, the retrozyme RNA can be replicated by Pol II as known for Pospiviroids, whereas in the chloroplast replication can depend on the RNA polymerase NEP, as known for Avsunviroids. In both cases, the replication products must then be transported to the cytoplasm for subsequent trafficking through plasmodesmata (PD) to neighboring cells and systemic leaves. It is likely that at this stage, the structured retrozyme RNA can be recognized by DCL proteins, which process it into small RNAs that can regulate the level of accumulation of the targeted mRNAs. Alternatively, the retrozyme circRNA may act as a template for protein expression or bind cellular miRNAs to act as a miRNA sponge.

As previously highlighted, there is a number of common structural and biological properties shared by HHR-containing viroids and plant retrozymes [154]. The similarities between HHR-containing viroids and retrozymes indicate that Avsunviroids may have evolved from plant retrozymes through genome escape [15,211]. In contrast to retrozymes, viroids have been the subject of significantly more extensive biological investigation to date [91]. In light of the available data on viroids and the potential origin of viroids from retrozymes, we propose the following model for presumed retrozyme functioning in plants (Figure 2). In response to stress or at a certain stage of development, the activation of the retrozyme promoter results in the appearance of the corresponding transcript. Subsequently, the retrozyme RNA is circularized through HHRs, and replication then proceeds by a rolling circle mechanism [12]. Replication is thought to occur through the activity of cellular RNA polymerase, as has been demonstrated in the case of viroids [91]. However, the compartment in which retrozyme replication occurs remains to be identified. In analogy with Avsunviroids, it may be assumed that the retrozyme RNA transcribed in the nucleus and processed by the ribozyme has 5′-hydroxyl and 2′,3′-cyclic phosphate ends [121,122]. It is possible that this RNA is transported to the cytoplasm and then to the chloroplasts, where it is ligated to form circular retrozyme RNAs and replicated by the chloroplast RNA polymerase NEP [118]. Further studies are needed to understand whether translation on the retrozyme circRNA template can occur in chloroplasts or the cytoplasm and what the function of the potential translation product might be. In the retrozyme lifecycle, replication products must be transported to neighboring cells and systemic leaves. It is likely that, in the cytoplasm, the structured retrozyme RNA can be recognized by DCL proteins as a substrate and processed into small RNAs that can regulate the level of accumulation of targeted mRNAs in a manner similar to that observed with viroid-derived sRNAs [212]. Alternatively, the retrozyme RNA may be able to bind cellular miRNAs and act as a miRNA sponge. In summary, the proposed model suggests that plants may use retrozyme RNA in two ways: first, to regulate processes in the cells where the retrozyme transcription has been activated, and second, to act as a long-distance messenger capable of inducing systemic effects.

## 5. Biotechnology of circRNAs

The progress made in the field of circRNA research has led to significant insights into the biogenesis and functions of these molecules in various systems. The accumulated data on the molecular mechanisms of action of circRNAs have enabled the development of promising biotechnological approaches, which are discussed below.

### 5.1. Modulation of Gene Expression

Since circRNAs are involved in the regulation of gene expression in plants, it is tempting to modify existing plant-encoded circRNAs or to engineer artificial circRNAs to influence circRNA-dependent regulatory pathways with the ultimate goal of generating plants with improved traits for agricultural needs. An example of such an approach is a recent work on several rice circRNAs, in which CRISPR-Cas9 editing of the rice genome was used to generate null mutants of individual circRNAs. Analysis of the resulting plant lines revealed that knockouts of circRNAs led to discernible phenotypic effects. In particular, the null mutant of rice circRNA Os05circ02465 exhibited high salt tolerance, while the Os06circ02797 mutant was characterized by faster seedling growth and higher chlorophyll A/B content compared to the wild-type plants [213]. In the latter case, the effect of circRNA knockout was associated with a higher accumulation level of Os-miR408 and concomitant downregulation of Os-miR408-targeted genes, suggesting that Os06circ02797 may serve as a sponge for Os-miR408, thereby participating in the regulation of gene expression by Os-miR408 [213]. Thus, manipulating the expression of circRNAs is shown to be an effective method for obtaining plant lines with altered traits and has therefore been introduced as a potential general strategy for crop improvement [213]. However, it should be noted that in the rice circRNA null mutants studied, the expression levels of many—up to hundreds—genes that direct the synthesis of mRNAs and small RNAs appeared to be altered [213], indicating that introducing circRNA mutations into plant genomes can have pleiotropic effects, most of which are unpredictable at the current level of understanding of circRNA functions.

Another approach to modulating gene expression in plants may involve replication-competent circRNAs, viroids or retrozymes, which can be engineered to carry foreign sequences and then used to infect plants. For example, the circRNA genome of the ELVd was used to develop a vector for post-transcriptional gene silencing in plants [214]. This approach was named viroid-induced gene silencing (VdIGS), in analogy to virus-induced gene silencing (VIGS), the method routinely used to downregulate gene expression in plants via RNA interference [215]. The major advantage of ELVd-based VdIGS over VIGS is the absence of severe symptoms resulting from viral infection and the absence of effects of virus-encoded proteins on the host cell. However, the VdIGS method has two major limitations, namely a small insert size and the need to preserve the secondary structure of the viroid RNA [214], due to the limited size of the viroid genome and the key function of the secondary/tertiary RNA structure in viroid RNA replication and transport [216]. Nevertheless, the approach to gene silencing in plants based on replication-competent circRNAs appeared to be valid, and further improvement of this technology may pave the way for its wider use.

### 5.2. RNA and Protein Production Systems

There is a growing need for new and efficient approaches to recombinant RNA production. However, the short half-life of the desired RNA often complicates the manufacturing and application processes. One possible strategy to address this issue is to develop a methodology to improve RNA stability. The desired RNA can be incorporated into stable scaffolds [217]. Incorporation of chimeric RNAs into such scaffolds may ensure stability and retention of function. Viroid genomic RNA, which has evolved into an extremely stable molecule, can also be used as a scaffold for engineering RNA production systems. Such a system has been developed based on ELVd. Chimeric aptamers and other structured RNAs were produced at high levels upon coexpression of recombinant ELVd with tRNA ligase that mediates viroid RNA circularization in *E. coli* [218]. However, RNA derived from an infectious agent may not be suitable for commercial use. To address this issue, a double-intron strategy was developed to produce recombinant circular dsRNA, in which the ELVd scaffold is removed [219]. This approach was effectively used to generate circular dsRNAs consisting of an 83 bp dsRNA, which was flanked only by exon fragments, specific to the DvSSJ1 gene of western cornworm (*Diabrotica virgifera*), one of the most damaging insect pests of cultivated maize [220], and exhibited insecticidal activity due to DvSSJ1 malfunction caused by RNA interference [219].

One of the main advantages of circRNAs over mRNAs is their potentially higher stability, which is an important factor in many applications [10]. To achieve increased protein production from full-length RNA messages in eukaryotic cells, engineered circRNAs have been successfully used [9]. In this case, RNA circularization was facilitated by the permuted intron−exon (PIE) splicing strategy, in which permuted group I intron precursor RNAs containing end-to-end fused exons that interrupt half-intron sequences are capable of self-splicing activity [221]. It has been demonstrated that the translation of circRNAs can be directed by IRES elements [181]. Therefore, to allow the protein translation on the template of circRNAs, a full-length IRES of the encephalomyocarditis virus was inserted upstream of an ORF encoding the luciferase. This approach demonstrated that circRNAs can produce higher levels of protein for longer periods of time than the linear RNAs used in this study, proving evidence that circRNAs have the potential to be used as an alternative to mRNAs for stable protein expression in different systems [9].

Following this pioneering study, Chen and colleagues have developed a highly effective circRNA-based platform for protein expression. Comprehensive optimization of the engineered circRNA (vector topology, 5′ and 3′ untranslated regions, IRES, and synthetic aptamers recruiting translation initiation factors) resulted in a significant increase in protein yield, higher translation efficiency compared to mRNA in vitro, and more durable translation in vivo [182]. Notably, prolonged and increased antigen production was also observed for circRNA vaccines, suggesting that they may be a promising approach. However, their safety profile remains to be established [222,223].

Currently available data suggest that further research in the field of circRNA biology is needed to fully explore the potential of circRNAs for various applications. These include further optimization of protein translation with circRNAs and a comprehensive study of the translation efficiency, stability, and functions of circRNAs in different cell types, tissues, and organisms.

## 6. Conclusions

Although circRNAs were discovered decades ago, many aspects of their biological functions remain unclear. In recent years, the role of circRNAs has been reconsidered, and it is now recognized that they have important functions. Targeted knockout or overexpression of specific circRNAs has been shown to alter the transcriptome and physiology of plants. However, the functions and mechanisms of action of circRNAs remain poorly understood, and only a limited number of studies have been conducted. The available information suggests that circRNAs are capable of acting as miRNA sponges, encoding functional proteins and forming R-loops with genomic DNA. Further research may reveal additional mechanisms associated with the ability of circRNAs to participate in systemic regulation in plants. For example, the detection of circRNAs in the apoplastic fluid of *A. thaliana* suggests that they are capable of long-distance transport [39]; however, the mechanism of circRNA transport and the targets of systemic regulation by circRNA remain to be determined.

Despite the extensive studies in plant viroid biology, there are still gaps in this area of research. In particular, the functional significance of the ORFs found in viroid RNAs needs to be further investigated. It is still unknown whether these ORFs getting translated and under what conditions this may occur. In addition, while RNA structural elements that can direct viroid transport between different cell types have been identified, the proteins that facilitate such transport remain undiscovered. In contrast to viroids, retrozymes have only recently been identified, and their biology remains to be understood. More specifically, it is not yet clear where and in response to what stimuli retrozymes are expressed, where their replication occurs, whether they can encode proteins, and what function the circRNA of retrozymes in plants may have. These knowledge gaps in the viroid and retrozyme biology remain the subject of ongoing and future research.

On the other hand, the involvement of circRNAs in the regulation of gene expression, as well as their stability, makes circRNAs an attractive tool in plant biotechnology, and recent pioneering studies have demonstrated their potential in this field. As data on the biology of circRNAs continue to accumulate, new circRNA-based biotechnological approaches with potential applications in research, medicine, and agriculture are likely to emerge.

## Data Availability

Not applicable.

## References

[1] Carninci P., Kasukawa T., Katayama S., Gough J., Frith M.C., Maeda N., Oyama R., Ravasi T., Lenhard B., Wells C. (2005). Molecular Biology: The Transcriptional Landscape of the Mammalian Genome. Science.

[2] Mattick J.S., Amaral P.P., Carninci P., Carpenter S., Chang H.Y., Chen L.L., Chen R., Dean C., Dinger M.E., Fitzgerald K.A. (2023). Long Non-Coding RNAs: Definitions, Functions, Challenges and Recommendations. Nat. Rev. Mol. Cell Biol..

[3] Wierzbicki A.T., Blevins T., Swiezewski S. (2021). Long Noncoding RNAs in Plants. Annu. Rev. Plant Biol..

[4] Cocquerelle C., Mascrez B., Hétuin D., Bailleul B. (1993). Mis-Splicing Yields Circular RNA Molecules. FASEB J..

[5] Ashwal-Fluss R., Meyer M., Pamudurti N.R., Ivanov A., Bartok O., Hanan M., Evantal N., Memczak S., Rajewsky N., Kadener S. (2014). CircRNA Biogenesis Competes with Pre-MRNA Splicing. Mol. Cell.

[6] Diener T.O. (1971). Potato Spindle Tuber “Virus”. IV. A Replicating, Low Molecular Weight RNA. Virology.

[7] Sogo J.M., Koller T., Diener T.O. (1973). Potato Spindle Tuber Viroid. X. Visualization and Size Determination by Electron Microscopy. Virology.

[8] Liu R., Ma Y., Guo T., Li G. (2023). Identification, Biogenesis, Function, and Mechanism of Action of Circular RNAs in Plants. Plant Commun..

[9] Wesselhoeft R.A., Kowalski P.S., Anderson D.G. (2018). Engineering Circular RNA for Potent and Stable Translation in Eukaryotic Cells. Nat. Commun..

[10] Enuka Y., Lauriola M., Feldman M.E., Sas-Chen A., Ulitsky I., Yarden Y. (2016). Circular RNAs Are Long-Lived and Display Only Minimal Early Alterations in Response to a Growth Factor. Nucleic Acids Res.

[11] Yang Y., Fan X., Mao M., Song X., Wu P., Zhang Y., Jin Y., Yang Y., Chen L.L., Wang Y. (2017). Extensive Translation of Circular RNAs Driven by N6-Methyladenosine. Cell Res..

[12] Cervera A., Urbina D., de la Peña M. (2016). Retrozymes Are a Unique Family of Non-Autonomous Retrotransposons with Hammerhead Ribozymes That Propagate in Plants Through Circular RNAs. Genome Biol..

[13] Lezzhov A.A., Tolstyko E.A., Atabekova A.K., Chergintsev D.A., Morozov S.Y., Solovyev A.G. (2022). In-Plant Persistence and Systemic Transport of Nicotiana Benthamiana Retrozyme RNA. Int. J. Mol. Sci..

[14] de la Peña M., Cervera A. (2017). Circular RNAs with Hammerhead Ribozymes Encoded in Eukaryotic Genomes: The Enemy at Home. RNA Biol..

[15] Marquez-Molins J. (2024). Uncovered Diversity of Infectious Circular RNAs: A New Paradigm for the Minimal Parasites?. NPJ Viruses.

[16] Marquez-Molins J., Navarro J.A., Seco L.C., Pallas V., Gomez G. (2021). Might Exogenous Circular RNAs Act as Protein-Coding Transcripts in Plants?. RNA Biol..

[17] AbouHaidar M.G., Venkataraman S., Golshani A., Liu B., Ahmad T. (2014). Novel Coding, Translation, and Gene Expression of a Replicating Covalently Closed Circular RNA of 220 nt. Proc. Natl. Acad. Sci. USA.

[18] Pasman Z., Been M.D., Garcia-Blanco M.A. (1996). Exon Circularization in Mammalian Nuclear Extracts. RNA.

[19] Schindewolf C., Braun S., Domdey H. (1996). In Vitro Generation of a Circular Exon from a Linear Pre-MRNA Transcript. Nucleic Acids Res..

[20] Braun S., Domdey H., Wiebauer K. (1996). Inverse Splicing of a Discontinuous Pre-MRNA Intron Generates a Circular Exon in a HeLa Cell Nuclear Extract. Nucleic Acids Res..

[21] Barrett S.P., Wang P.L., Salzman J. (2015). Circular RNA Biogenesis Can Proceed through an Exon-Containing Lariat Precursor. Elife.

[22] Liao X., Li X.J., Zheng G.T., Chang F.R., Fang L., Yu H., Huang J., Zhang Y.F. (2022). Mitochondrion-Encoded Circular RNAs Are Widespread and Translatable in Plants. Plant Physiol..

[23] Liu S., Wang Q., Li X., Wang G., Wan Y. (2019). Detecting of Chloroplast Circular RNAs in Arabidopsis Thaliana. Plant Signal. Behav..

[24] Zhang X.O., Wang H.B., Zhang Y., Lu X., Chen L.L., Yang L. (2014). Complementary Sequence-Mediated Exon Circularization. Cell.

[25] Kramer M.C., Liang D., Tatomer D.C., Gold B., March Z.M., Cherry S., Wilusz J.E. (2015). Combinatorial Control of Drosophila Circular RNA Expression by Intronic Repeats, HnRNPs, and SR Proteins. Genes Dev..

[26] Liang D., Wilusz J.E. (2014). Short Intronic Repeat Sequences Facilitate Circular RNA Production. Genes Dev..

[27] Payer L.M., Steranka J.P., Ardeljan D., Walker J., Fitzgerald K.C., Calabresi P.A., Cooper T.A., Burns K.H. (2019). Alu Insertion Variants Alter MRNA Splicing. Nucleic Acids Res..

[28] Chen L., Zhang P., Fan Y., Lu Q., Li Q., Yan J., Muehlbauer G.J., Schnable P.S., Dai M., Li L. (2018). Circular RNAs Mediated by Transposons Are Associated with Transcriptomic and Phenotypic Variation in Maize. New Phytol..

[29] Conn S.J., Pillman K.A., Toubia J., Conn V.M., Salmanidis M., Phillips C.A., Roslan S., Schreiber A.W., Gregory P.A., Goodall G.J. (2015). The RNA Binding Protein Quaking Regulates Formation of CircRNAs. Cell.

[30] Beuck C., Qu S., Fagg W.S., Ares M., Williamson J.R. (2012). Structural Analysis of the Quaking Homodimerization Interface. J. Mol. Biol..

[31] Lai X., Bazin J., Webb S., Crespi M., Zubieta C., Conn S.J. (2018). CircRNAs in Plants. Adv. Exp. Med. Biol..

[32] Zhang J., Zhang X., Li C., Yue L., Ding N., Riordan T., Yang L., Li Y., Jen C., Lin S. (2019). Circular RNA Profiling Provides Insights into Their Subcellular Distribution and Molecular Characteristics in HepG2 Cells. RNA Biol..

[33] Sadhukhan S., Sinha T., Dey S., Panda A.C. (2024). Subcellular Localization of Circular RNAs: Where and Why. Biochem. Biophys. Res. Commun..

[34] Liu C.X., Chen L.L. (2022). Circular RNAs: Characterization, Cellular Roles, and Applications. Cell.

[35] Hon K.W., Ab-Mutalib N.S., Abdullah N.M.A., Jamal R., Abu N. (2019). Extracellular Vesicle-Derived Circular RNAs Confers Chemoresistance in Colorectal Cancer. Sci. Rep..

[36] Guo C., Lv H., Bai Y., Guo M., Li P., Tong S., He K. (2023). Circular RNAs in Extracellular Vesicles: Promising Candidate Biomarkers for Schizophrenia. Front. Genet..

[37] Lee H., Hong R., Jin Y. (2024). Altered Circular RNA Expressions in Extracellular Vesicles from Bronchoalveolar Lavage Fluids in Mice after Bacterial Infections. Front. Immunol..

[38] Li Q., Geng S., Yuan H., Li Y., Zhang S., Pu L., Ge J., Niu X., Li Y., Jiang H. (2019). Circular RNA Expression Profiles in Extracellular Vesicles from the Plasma of Patients with Pancreatic Ductal Adenocarcinoma. FEBS Open Bio.

[39] Zand Karimi H., Baldrich P., Rutter B.D., Borniego L., Zajt K.K., Meyers B.C., Innes R.W. (2022). Arabidopsis Apoplastic Fluid Contains SRNA- and Circular RNA–Protein Complexes That Are Located Outside Extracellular Vesicles. Plant Cell.

[40] Hwang H.J., Kim Y.K. (2024). Molecular Mechanisms of Circular RNA Translation. Exp. Mol. Med..

[41] Wang Y., Wang Z. (2015). Efficient Backsplicing Produces Translatable Circular MRNAs. RNA.

[42] Choe J., Lin S., Zhang W., Liu Q., Wang L., Ramirez-Moya J., Du P., Kim W., Tang S., Sliz P. (2018). MRNA Circularization by METTL3–EIF3h Enhances Translation and Promotes Oncogenesis. Nature.

[43] Shi H., Wang X., Lu Z., Zhao B.S., Ma H., Hsu P.J., Liu C., He C. (2017). YTHDF3 Facilitates Translation and Decay of N 6-Methyladenosine-Modified RNA. Cell Res..

[44] Han Y., Li X., Yan Y., Duan M.H., Xu J.H. (2020). Identification, Characterization, and Functional Prediction of Circular RNAs in Maize. Mol. Genet. Genom..

[45] Wang Y., Wang H., Xi F., Wang H., Han X., Wei W., Zhang H., Zhang Q., Zheng Y., Zhu Q. (2020). Profiling of Circular RNA N6-Methyladenosine in Moso Bamboo (*Phyllostachys edulis*) Using Nanopore-Based Direct RNA Sequencing. J. Integr. Plant Biol..

[46] Chen L., Ding X., Zhang H., He T., Li Y., Wang T., Li X., Jin L., Song Q., Yang S. (2018). Comparative Analysis of Circular RNAs between Soybean Cytoplasmic Male-Sterile Line NJCMS1A and Its Maintainer NJCMS1B by High-Throughput Sequencing. BMC Genom..

[47] Sun P., Li G. (2019). CircCode: A Powerful Tool for Identifying CircRNA Coding Ability. Front. Genet..

[48] Li S., Li X., Xue W., Zhang L., Yang L.Z., Cao S.M., Lei Y.N., Liu C.X., Guo S.K., Shan L. (2021). Screening for Functional Circular RNAs Using the CRISPR–Cas13 System. Nat. Methods.

[49] Singh S., Sinha T., Panda A.C. (2024). Regulation of MicroRNA by Circular RNA. Wiley Interdiscip. Rev. RNA.

[50] Hansen T.B., Jensen T.I., Clausen B.H., Bramsen J.B., Finsen B., Damgaard C.K., Kjems J. (2013). Natural RNA Circles Function as Efficient MicroRNA Sponges. Nature.

[51] Xu Y., Leng K., Yao Y., Kang P., Liao G., Han Y., Shi G., Ji D., Huang P., Zheng W. (2021). A Novel Circular RNA, Circ-CCAC1, Contributes to CCA Progression, Induces Angiogenesis, and Disrupts Vascular Endothelial Barriers. Hepatology.

[52] Abdelmohsen K., Panda A.C., Munk R., Grammatikakis I., Dudekula D.B., De S., Kim J., Noh J.H., Kim K.M., Martindale J.L. (2017). Identification of HuR Target Circular RNAs Uncovers Suppression of PABPN1 Translation by CircPABPN1. RNA Biol..

[53] Huang S., Li X., Zheng H., Si X., Li B., Wei G., Li C., Chen Y., Chen Y., Liao W. (2019). Loss of Super-Enhancer-Regulated CircRNA Nfix Induces Cardiac Regeneration After Myocardial Infarction in Adult Mice. Circulation.

[54] Du W.W., Fang L., Yang W., Wu N., Awan F.M., Yang Z., Yang B.B. (2017). Induction of Tumor Apoptosis Through a Circular RNA Enhancing Foxo3 Activity. Cell Death Differ..

[55] Huang A., Zheng H., Wu Z., Chen M., Huang Y. (2020). Circular RNA-Protein Interactions: Functions, Mechanisms, and Identification. Theranostics.

[56] Li Z., Huang C., Bao C., Chen L., Lin M., Wang X., Zhong G., Yu B., Hu W., Dai L. (2015). Exon-Intron Circular RNAs Regulate Transcription in the Nucleus. Nat. Struct. Mol. Biol..

[57] Conn V.M., Hugouvieux V., Nayak A., Conos S.A., Capovilla G., Cildir G., Jourdain A., Tergaonkar V., Schmid M., Zubieta C. (2017). A CircRNA from SEPALLATA3 Regulates Splicing of Its Cognate MRNA through R-Loop Formation. Nat. Plants.

[58] Gu Y., Wang Y., He L., Zhang J., Zhu X., Liu N., Wang J., Lu T., He L., Tian Y. (2021). Circular RNA CircIPO11 Drives Self-Renewal of Liver Cancer Initiating Cells via Hedgehog Signaling. Mol. Cancer.

[59] Babaei S., Singh M.B., Bhalla P.L. (2021). Circular RNAs Repertoire and Expression Profile During *Brassica rapa* Pollen Development. Int. J. Mol. Sci..

[60] Xu H., Chen B., Zhao Y., Guo Y., Liu G., Li R., Zeisler-Diehl V.V., Chen Y., He X., Schreiber L. (2022). Non-Coding RNA Analyses of Seasonal Cambium Activity in Populus Tomentosa. Cells.

[61] Chen X., Xu X., Zhang S., Munir N., Zhu C., Zhang Z., Chen Y., Xuhan X., Lin Y., Lai Z. (2022). Genome-Wide Circular RNA Profiling and Competing Endogenous RNA Regulatory Network Analysis Provide New Insights into the Molecular Mechanisms Underlying Early Somatic Embryogenesis in Dimocarpus Longan Lour. Tree Physiol..

[62] Li S., Wang J., Ren G. (2024). CircRNA: An Emerging Star in Plant Research: A Review. Int. J. Biol. Macromol..

[63] Xiang L., Cai C., Cheng J., Wang L., Wu C., Shi Y., Luo J., He L., Deng Y., Zhang X. (2018). Identification of CircularRNAs and Their Targets in Gossypium Under Verticillium Wilt Stress Based on RNA-Seq. PeerJ.

[64] Ghorbani A., Izadpanah K., Peters J.R., Dietzgen R.G., Mitter N. (2018). Detection and Profiling of Circular RNAs in Uninfected and Maize Iranian Mosaic Virus-Infected Maize. Plant Sci..

[65] Wang J., Yang Y., Jin L., Ling X., Liu T., Chen T., Ji Y., Yu W., Zhang B. (2018). Re-Analysis of Long Non-Coding RNAs and Prediction of CircRNAs Reveal Their Novel Roles in Susceptible Tomato Following TYLCV Infection. BMC Plant Biol..

[66] Wang Z., Liu Y., Li D., Li L., Zhang Q., Wang S., Huang H. (2017). Identification of Circular RNAs in Kiwifruit and Their Species-Specific Response to Bacterial Canker Pathogen Invasion. Front. Plant Sci..

[67] Zhou R., Zhu Y., Zhao J., Fang Z., Wang S., Yin J., Chu Z., Ma D. (2018). Transcriptome-Wide Identification and Characterization of Potato Circular RNAs in Response to *Pectobacterium carotovorum* Subspecies Brasiliense Infection. Int. J. Mol. Sci..

[68] Fu X.-Z., Zhang X.-Y., Qiu J.-Y., Zhou X., Yuan M., He Y.-Z., Chun C.-P., Cao L., Ling L.-L., Peng L.-Z. (2019). Whole-Transcriptome RNA Sequencing Reveals the Global Molecular Responses and CeRNA Regulatory Network of MRNAs, LncRNAs, MiRNAs and CircRNAs in Response to Copper Toxicity in Ziyang Xiangcheng (*Citrus junos* Sieb. Ex Tanaka). BMC Plant Biol..

[69] Yin Z., Zhao Q., Lv X., Zhang X., Wu Y. (2024). Circular RNA Ath-Circ032768, a Competing Endogenous RNA, Response the Drought Stress by Targeting MiR472-RPS5 Module. Plant Biol..

[70] Wang W., Wang J., Wei Q., Li B., Zhong X., Hu T., Hu H., Bao C. (2019). Transcriptome-Wide Identification and Characterization of Circular RNAs in Leaves of Chinese Cabbage (*Brassica rapa* L. ssp. *Pekinensis*) in Response to Calcium Deficiency-Induced Tip-Burn. Sci. Rep..

[71] Lv L., Yu K., Lü H., Zhang X., Liu X., Sun C., Xu H., Zhang J., He X., Zhang D. (2020). Transcriptome-Wide Identification of Novel Circular RNAs in Soybean in Response to Low-Phosphorus Stress. PLoS ONE.

[72] Gao Z., Li J., Luo M., Li H., Chen Q., Wang L., Song S., Zhao L., Xu W., Zhang C. (2019). Characterization and Cloning of Grape Circular RNAs Identified the Cold Resistance-Related vv-Circats1. Plant Physiol..

[73] García-Muse T., Aguilera A. (2019). R Loops: From Physiological to Pathological Roles. Cell.

[74] Xu W., Xu H., Li K., Fan Y., Liu Y., Yang X., Sun Q. (2017). The R-Loop Is a Common Chromatin Feature of the Arabidopsis Genome. Nat. Plants.

[75] Liu Y., Su H., Zhang J., Liu Y., Feng C., Han F. (2020). Back-Spliced RNA from Retrotransposon Binds to Centromere and Regulates Centromeric Chromatin Loops in Maize. PLoS Biol..

[76] Thomas L.F., Sætrom P. (2014). Circular RNAs Are Depleted of Polymorphisms at MicroRNA Binding Sites. Bioinformatics.

[77] Qi D., Dubiella U., Kim S.H., Isaiah Sloss D., Dowen R.H., Dixon J.E., Innes R.W. (2014). Recognition of the Protein Kinase AVRPPHB SUSCEPTIBLE1 by the Disease Resistance Protein RESISTANCE TO PSEUDOMONAS SYRINGAE5 Is Dependent on S-Acylation and an Exposed Loop in AVRPPHB SUSCEPTIBLE. Plant Physiol..

[78] Capelari É.F., da Fonseca G.C., Guzman F., Margis R. (2019). Circular and Micro RNAs from *Arabidopsis thaliana* Flowers Are Simultaneously Isolated from AGO-IP Libraries. Plants.

[79] Navarro B., Flores R. (1997). Chrysanthemum Chlorotic Mottle Viroid: Unusual Structural Properties of a Subgroup of Self-Cleaving Viroids with Hammerhead Ribozymes. Proc. Natl. Acad. Sci. USA.

[80] Fadda Z., Daròs J.A., Flores R., Duran-Vila N. (2003). Identification in eggplant of a variant of citrus exocortis viroid (CEVd) with a 96 nucleotide duplication in the right terminal region of the rod-like secondary structure. Virus Res..

[81] Flores R., Hernández C., Martínez De Alba A.E., Daròs J.A., Di Serio F. (2005). Viroids and Viroid-Host Interactions. Annu. Rev. Phytopathol..

[82] Flores R., Minoia S., López-Carrasco A., Delgado S., Martínez de Alba A.E., Kalantidis K. (2017). Viroid Replication. Viroids and Satellites.

[83] Venkataraman S., Badar U., Shoeb E., Hashim G., Abouhaidar M., Hefferon K. (2021). An Inside Look into Biological Miniatures: Molecular Mechanisms of Viroids. Int. J. Mol. Sci..

[84] Hao J., Ma J., Wang Y. (2024). Understanding Viroids, Endogenous Circular RNAs, and Viroid-like RNAs in the Context of Biogenesis. PLOS Pathog..

[85] Ortolá B., Daròs J.A. (2023). Viroids: Non-Coding Circular RNAs Able to Autonomously Replicate and Infect Higher Plants. Biology.

[86] Steger G., Perreault J.P. (2016). Structure and Associated Biological Functions of Viroids. Adv. Virus Res..

[87] Sano T., Candresse T., Hammond R.W., Diener T.O., Owens R.A. (1992). Identification of Multiple Structural Domains Regulating Viroid Pathogenicity. Proc. Natl. Acad. Sci. USA.

[88] Flores R., Serra P., Minoia S., Di Serio F., Navarro B. (2012). Viroids: From Genotype to Phenotype Just Relying on RNA Sequence and Structural Motifs. Front. Microbiol..

[89] Zhong X., Leontis N., Qian S., Itaya A., Qi Y., Boris-Lawrie K., Ding B. (2006). Tertiary Structural and Functional Analyses of a Viroid RNA Motif by Isostericity Matrix and Mutagenesis Reveal Its Essential Role in Replication. J. Virol..

[90] Wassenegger M., Spieker R.L., Thalmeir S., Gast F.U., Riedel L., Sänger H.L. (1996). A Single Nucleotide Substitution Converts Potato Spindle Tuber Viroid (PSTVd) from a Noninfectious to an Infectious RNA for Nicotiana Tabacum. Virology.

[91] Navarro B., Flores R., Di Serio F. (2021). Advances in Viroid-Host Interactions. Annu. Rev. Virol..

[92] Wüsthoff K.P., Steger G. (2022). Conserved Motifs and Domains in Members of Pospiviroidae. Cells.

[93] Flores R., Navarro B., Serra P., Di Serio F. (2022). A Scenario for the Emergence of Protoviroids in the RNA World and for Their Further Evolution into Viroids and Viroid-like RNAs by Modular Recombinations and Mutations. Virus Evol..

[94] Chiumenti M., Navarro B., Candresse T., Flores R., Di Serio F. (2021). Reassessing Species Demarcation Criteria in Viroid Taxonomy by Pairwise Identity Matrices. Virus Evol..

[95] Carbonell A., De la Peña M., Flores R., Gago S. (2006). Effects of the Trinucleotide Preceding the Self-Cleavage Site on Eggplant Latent Viroid Hammerheads: Differences in Co- and Post-Transcriptional Self-Cleavage May Explain the Lack of Trinucleotide AUC in Most Natural Hammerheads. Nucleic Acids Res..

[96] Flores R., Delgado S., Gas M.E., Carbonell A., Molina D., Gago S., De La Peña M. (2004). Viroids: The Minimal Non-Coding RNAs with Autonomous Replication. FEBS Lett..

[97] Zhang Y., Nie Y., Wang L., Wu J. (2024). Viroid Replication, Movement, and the Host Factors Involved. Microorganisms.

[98] Woo Y.M., Itaya A., Owens R.A., Tang L., Hammond R.W., Chou H.C., Lai M.M.C., Ding B. (1999). Characterization of Nuclear Import of Potato Spindle Tuber Viroid RNA in Permeabilized Protoplasts. Plant J..

[99] Gozmanova M., Denti M.A., Minkov I.N., Tsagris M., Tabler M. (2003). Characterization of the RNA Motif Responsible for the Specific Interaction of Potato Spindle Tuber Viroid RNA (PSTVd) and the Tomato Protein Virp1. Nucleic Acids Res..

[100] Kalantidis K., Denti M.A., Tzortzakaki S., Marinou E., Tabler M., Tsagris M. (2007). Virp1 Is a Host Protein with a Major Role in Potato Spindle Tuber Viroid Infection in Nicotiana Plants. J. Virol..

[101] Ma J., Dissanayaka Mudiyanselage S.D., Park W.J., Wang M., Takeda R., Liu B., Wang Y. (2022). A Nuclear Import Pathway Exploited by Pathogenic Noncoding RNAs. Plant Cell.

[102] Maniataki E., Tabler M., Tsagris M. (2003). Viroid RNA Systemic Spread May Depend on the Interaction of a 71-Nucleotide Bulged Hairpin with the Host Protein VirP1. RNA.

[103] Martínez de Alba A.E., Sägesser R., Tabler M., Tsagris M. (2003). A Bromodomain-Containing Protein from Tomato Specifically Binds Potato Spindle Tuber Viroid RNA in Vitro and in Vivo. J. Virol..

[104] Chaturvedi S., Kalantidis K., Rao A.L.N. (2014). A Bromodomain-Containing Host Protein Mediates the Nuclear Importation of a Satellite RNA of Cucumber Mosaic Virus. J. Virol..

[105] Abraitiene A., Zhao Y., Hammond R. (2008). Nuclear Targeting by Fragmentation of the Potato Spindle Tuber Viroid Genome. Biochem. Biophys. Res. Commun..

[106] Dissanayaka Mudiyanselage S.D., Ma J., Pechan T., Pechanova O., Liu B., Wang Y. (2022). A Remodeled RNA Polymerase II Complex Catalyzing Viroid RNA-Templated Transcription. PLoS Pathog..

[107] Dissanayaka Mudiyanselage S.D., Qu J., Tian N., Jiang J., Wang Y. (2018). Potato Spindle Tuber Viroid RNA-Templated Transcription: Factors and Regulation. Viruses.

[108] Qi Y., Ding B. (2003). Differential Subnuclear Localization of RNA Strands of Opposite Polarity Derived from an Autonomously Replicating Viroid. Plant Cell.

[109] Gas M.E., Hernández C., Flores R., Daròs J.A. (2007). Processing of Nuclear Viroids In Vivo: An Interplay between RNA Conformations. PLoS Pathog..

[110] Gas M.-E., Molina-Serrano D., Hernández C., Flores R., Daròs J.-A. (2008). Monomeric Linear RNA of Citrus Exocortis Viroid Resulting from Processing In Vivo Has 5′-Phosphomonoester and 3′-Hydroxyl Termini: Implications for the RNase and RNA Ligase Involved in Replication. J. Virol..

[111] Baumstark T., Schröder A.R.W., Riesner D. (1997). Viroid Processing: Switch from Cleavage to Ligation Is Driven by a Change from a Tetraloop to a Loop E Conformation. EMBO J..

[112] Nohales M.-Á., Molina-Serrano D., Flores R., Daròs J.-A. (2012). Involvement of the Chloroplastic Isoform of TRNA Ligase in the Replication of Viroids Belonging to the Family Avsunviroidae. J. Virol..

[113] Bonfiglioli R.G., McFadden G.I., Symons R.H. (1994). In Situ Hybridization Localizes Avocado Sunblotch Viroid on Chloroplast Thylakoid Membranes and Coconut Cadang Cadang Viroid in the Nucleus. Plant J..

[114] Gómez G., Pallás V. (2010). Noncoding RNA Mediated Traffic of Foreign MRNA into Chloroplasts Reveals a Novel Signaling Mechanism in Plants. PLoS ONE.

[115] Gómez G., Pallás V. (2010). Can the Import of MRNA into Chloroplasts Be Mediated by a Secondary Structure of a Small Non-Coding RNA?. Plant Signal. Behav..

[116] Gómez G., Pallas V. (2012). Studies on Subcellular Compartmentalization of Plant Pathogenic Noncoding RNAs Give New Insights into the Intracellular RNA-Traffic Mechanisms. Plant Physiol..

[117] Martínez F., Marqués J., Salvador M.L., Darós J.A. (2009). Mutational Analysis of Eggplant Latent Viroid RNA Processing in Chlamydomonas Reinhardtii Chloroplast. J. Gen. Virol..

[118] Navarro J.A., Vera A., Flores R. (2000). A Chloroplastic RNA Polymerase Resistant to Tagetitoxin Is Involved in Replication of Avocado Sunblotch Viroid. Virology.

[119] Tsagris E.M., de Alba Á.E.M., Gozmanova M., Kalantidis K. (2008). Viroids. Cell. Microbiol..

[120] Gago S., Elena S.F., Flores R., Sanjuán R. (2009). Extremely High Mutation Rate of a Hammerhead Viroid. Science.

[121] de la Peña M., Gago-Zachert S. (2022). A Life of Research on Circular RNAs and Ribozymes: Towards the Origin of Viroids, Deltaviruses and Life. Virus Res..

[122] De la Peña M., Gago S., Flores R. (2003). Peripheral Regions of Natural Hammerhead Ribozymes Greatly Increase Their Self-Cleavage Activity. EMBO J..

[123] Martick M., Scott W.G. (2006). Tertiary Contacts Distant from the Active Site Prime a Ribozyme for Catalysis. Cell.

[124] Khvorova A., Lescoute A., Westhof E., Jayasena S.D. (2003). Sequence Elements Outside the Hammerhead Ribozyme Catalytic Core Enable Intracellular Activity. Nat. Struct. Biol..

[125] De La Peña M., García-Robles I., Cervera A. (2017). The Hammerhead Ribozyme: A Long History for a Short RNA. Mol. A J. Synth. Chem. Nat. Prod. Chem..

[126] Cordero T., Ortolá B., Daròs J.A. (2018). Mutational Analysis of Eggplant Latent Viroid RNA Circularization by the Eggplant TRNA Ligase in *Escherichia coli*. Front. Microbiol..

[127] Tolstyko E.A., Lezzhov A.A., Morozov S.Y., Solovyev A.G. (2020). Phloem Transport of Structured RNAs: A Widening Repertoire of Trafficking Signals and Protein Factors. Plant Sci..

[128] Wu J., Leontis N.B., Zirbel C.L., Bisaro D.M., Ding B. (2019). A Three-Dimensional RNA Motif Mediates Directional Trafficking of Potato Spindle Tuber Viroid from Epidermal to Palisade Mesophyll Cells in *Nicotiana benthamiana*. PLoS Pathog..

[129] Takeda R., Petrov A.I., Leontis N.B., Ding B. (2011). A Three-Dimensional RNA Motif in Potato Spindle Tuber Viroid Mediates Trafficking from Palisade Mesophyll to Spongy Mesophyll in *Nicotiana benthamiana*. Plant Cell.

[130] Jiang D., Wang M., Li S. (2017). Functional Analysis of a Viroid RNA Motif Mediating Cell-to-Cell Movement in *Nicotiana benthamiana*. J. Gen. Virol..

[131] Qi Y., Pélissier T., Itaya A., Hunt E., Wassenegger M., Ding B. (2004). Direct Role of a Viroid RNA Motif in Mediating Directional RNA Trafficking Across a Specific Cellular Boundary. Plant Cell.

[132] Gómez G., Pallás V. (2001). Identification of an in Vitro Ribonucleoprotein Complex between a Viroid RNA and a Phloem Protein from Cucumber Plants. Mol. Plant. Microbe. Interact..

[133] Gómez G., Pallás V. (2004). A Long-Distance Translocatable Phloem Protein from Cucumber Forms a Ribonucleoprotein Complex In Vivo with Hop Stunt Viroid RNA. J. Virol..

[134] Walia Y., Dhir S., Zaidi A.A., Hallan V. (2015). Apple Scar Skin Viroid Naked RNA Is Actively Transmitted by the Whitefly *Trialeurodes vaporariorum*. RNA Biol..

[135] Gómez G., Torres H., Pallás V. (2005). Identification of Translocatable RNA-Binding Phloem Proteins from Melon, Potential Components of the Long-Distance RNA Transport System. Plant J..

[136] Solovyev A.G., Makarova S.S., Remizowa M.V., Lim H.S., Hammond J., Owens R.A., Kopertekh L., Schiemann J., Morozov S.Y. (2013). Possible Role of the Nt-4/1 Protein in Macromolecular Transport in Vascular Tissue. Plant Signal. Behav..

[137] Adkar-Purushothama C.R., Brosseau C., Gigu È Re T., Sano T., Moffett P., Perreaulta J.P. (2015). Small RNA Derived from the Virulence Modulating Region of the Potato Spindle Tuber Viroid Silences Callose Synthase Genes of Tomato Plants. Plant Cell.

[138] Lisón P., Tárraga S., López-Gresa P., Saurí A., Torres C., Campos L., Bellés J.M., Conejero V., Rodrigo I. (2013). A Noncoding Plant Pathogen Provokes Both Transcriptional and Posttranscriptional Alterations in Tomato. Proteomics.

[139] Dubé A., Bisaillon M., Perreault J.-P. (2009). Identification of Proteins from Prunus Persica That Interact with Peach Latent Mosaic Viroid. J. Virol..

[140] Lisón P., Vázquez-Prol F., Bardani I., Rodrigo I., Kryovrysanaki N., Kalantidis K. (2024). Viroids and Protein Translation. Fundamentals of Viroid Biology.

[141] Cottilli P., Belda-Palazón B., Adkar-Purushothama C.R., Perreault J.P., Schleiff E., Rodrigo I., Ferrando A., Lisón P. (2019). Citrus Exocortis Viroid Causes Ribosomal Stress in Tomato Plants. Nucleic Acids Res..

[142] Prol F.V., Márquez-Molins J., Rodrigo I., López-Gresa M.P., Bellés J.M., Gómez G., Pallás V., Lisón P. (2021). Symptom Severity, Infection Progression and Plant Responses in Solanum Plants Caused by Three Pospiviroids Vary with the Inoculation Procedure. Int. J. Mol. Sci..

[143] Katsarou K., Adkar-Purushothama C.R., Tassios E., Samiotaki M., Andronis C., Lisón P., Nikolaou C., Perreault J.P., Kalantidis K. (2022). Revisiting the Non-Coding Nature of Pospiviroids. Cells.

[144] Delgado S., Navarro B., Serra P., Gentit P., Cambra M.Á., Chiumenti M., De Stradis A., Di Serio F., Flores R. (2019). How Sequence Variants of a Plastid-Replicating Viroid with One Single Nucleotide Change Initiate Disease in Its Natural Host. RNA Biol..

[145] Navarro B., Gisel A., Rodio M.E., Delgado S., Flores R., Di Serio F. (2012). Small RNAs Containing the Pathogenic Determinant of a Chloroplast-Replicating Viroid Guide the Degradation of a Host MRNA as Predicted by RNA Silencing. Plant J..

[146] Bao S., Owens R.A., Sun Q., Song H., Liu Y., Eamens A.L., Feng H., Tian H., Wang M.B., Zhang R. (2019). Silencing of Transcription Factor Encoding Gene StTCP23 by Small RNAs Derived from the Virulence Modulating Region of Potato Spindle Tuber Viroid Is Associated with Symptom Development in Potato. PLoS Pathog..

[147] Adkar-Purushothama C.R., Iyer P.S., Perreault J.P. (2017). Potato Spindle Tuber Viroid Infection Triggers Degradation of Chloride Channel Protein CLC-b-like and Ribosomal Protein S3a-like MRNAs in Tomato Plants. Sci. Rep..

[148] Adkar-Purushothama C.R., Sano T., Perreault J.P. (2018). Viroid-Derived Small RNA Induces Early Flowering in Tomato Plants by RNA Silencing. Mol. Plant Pathol..

[149] Ramesh S.V., Yogindran S., Gnanasekaran P., Chakraborty S., Winter S., Pappu H.R. (2020). Virus and Viroid-Derived Small RNAs as Modulators of Host Gene Expression: Molecular Insights into Pathogenesis. Front. Microbiol..

[150] Martinez G., Castellano M., Tortosa M., Pallas V., Gomez G. (2014). A Pathogenic Non-Coding RNA Induces Changes in Dynamic DNA Methylation of Ribosomal RNA Genes in Host Plants. Nucleic Acids Res..

[151] Earley K.W., Pontvianne F., Wierzbicki A.T., Blevins T., Tucker S., Costa-Nunes P., Pontes O., Pikaard C.S. (2010). Mechanisms of HDA6-Mediated RRNA Gene Silencing: Suppression of Intergenic Pol II Transcription and Differential Effects on Maintenance versus SiRNA-Directed Cytosine Methylation. Genes Dev..

[152] Castellano M., Pallas V., Gomez G. (2016). A Pathogenic Long Noncoding RNA Redesigns the Epigenetic Landscape of the Infected Cells by Subverting Host Histone Deacetylase 6 Activity. New Phytol..

[153] Navarro B., Rubino L., Di Serio F. (2017). Small Circular Satellite RNAs. Viroids and Satellites.

[154] Lee B.D., Koonin E.V. (2022). Viroids and Viroid-like Circular RNAs: Do They Descend from Primordial Replicators?. Life.

[155] Vera A., Daròs J.-A., Flores R., Hernández C. (2000). The DNA of a Plant Retroviroid-like Element Is Fused to Different Sites in the Genome of a Plant Pararetrovirus and Shows Multiple Forms with Sequence Deletions. J. Virol..

[156] Balázs E., Hegedűs K., Divéki Z. (2022). The Unique Carnation Stunt-Associated Pararetroviroid. Virus Res..

[157] Peña M., Ceprián R., Cervera A. (2020). A Singular and Widespread Group of Mobile Genetic Elements: RNA Circles with Autocatalytic Ribozymes. Cells.

[158] De La Peña M., García-Robles I. (2010). Ubiquitous Presence of the Hammerhead Ribozyme Motif along the Tree of Life. RNA.

[159] Gao D., Chen J., Chen M., Meyers B.C., Jackson S. (2012). A Highly Conserved, Small LTR Retrotransposon That Preferentially Targets Genes in Grass Genomes. PLoS ONE.

[160] Witte C.P., Le Q.H., Bureau T., Kumar A. (2001). Terminal-Repeat Retrotransposons in Miniature (TRIM) Are Involved in Restructuring Plant Genomes. Proc. Natl. Acad. Sci. USA.

[161] Cervera A., De La Peña M. (2020). Small CircRNAs with Self-Cleaving Ribozymes Are Highly Expressed in Diverse Metazoan Transcriptomes. Nucleic Acids Res..

[162] Schostak N., Pyatkov K., Zelentsova E., Arkhipova I., Shagin D., Shagina I., Mudrik E., Blintsov A., Clark I., Finnegan D.J. (2008). Molecular Dissection of Penelope Transposable Element Regulatory Machinery. Nucleic Acids Res..

[163] Cervera A., De La Peña M. (2014). Eukaryotic Penelope-like Retroelements Encode Hammerhead Ribozyme Motifs. Mol. Biol. Evol..

[164] Gladyshev E.A., Arkhipova I.R. (2007). Telomere-Associated Endonuclease-Deficient Penelope-like Retroelements in Diverse Eukaryotes. Proc. Natl. Acad. Sci. USA.

[165] Cavrak V.V., Lettner N., Jamge S., Kosarewicz A., Bayer L.M., Mittelsten Scheid O. (2014). How a Retrotransposon Exploits the Plant’s Heat Stress Response for Its Activation. PLoS Genet..

[166] Wang S., Wang M., Ichino L., Boone B.A., Zhong Z., Papareddy R.K., Lin E.K., Yun J., Feng S., Jacobsen S.E. (2024). MBD2 Couples DNA Methylation to Transposable Element Silencing during Male Gametogenesis. Nat. Plants.

[167] Fultz D., Choudury S.G., Slotkin R.K. (2015). Silencing of Active Transposable Elements in Plants. Curr. Opin. Plant Biol..

[168] Feschotte C., Jiang N., Wessler S.R. (2002). Plant Transposable Elements: Where Genetics Meets Genomics. Nat. Rev. Genet..

[169] Arkhipova I.R. (2006). Distribution and Phylogeny of Penelope-like Elements in Eukaryotes. Syst. Biol..

[170] Arkhipova I.R., Pyatkov K.I., Meselson M., Evgen’ev M.B. (2003). Retroelements Containing Introns in Diverse Invertebrate Taxa. Nat. Genet..

[171] Lünse C.E., Weinberg Z., Breaker R.R. (2017). Numerous Small Hammerhead Ribozyme Variants Associated with Penelope-like Retrotransposons Cleave RNA as Dimers. RNA Biol..

[172] Diener T.O. (2016). Viroids: “Living Fossils” of Primordial RNAs?. Biol. Direct.

[173] Flores R., Gago-Zachert S., Serra P., Sanjuán R., Elena S.F. (2014). Viroids: Survivors from the RNA World?. Annu. Rev. Microbiol..

[174] Catalán P., Elena S.F., Cuesta J.A., Manrubia S. (2019). Parsimonious Scenario for the Emergence of Viroid-Like Replicons De Novo. Viruses.

[175] López-Carrasco A., Ballesteros C., Sentandreu V., Delgado S., Gago-Zachert S., Flores R., Sanjuán R. (2017). Different Rates of Spontaneous Mutation of Chloroplastic and Nuclear Viroids as Determined by High-Fidelity Ultra-Deep Sequencing. PLoS Pathog..

[176] Daròs J.A., Flores R. (1995). Identification of a Retroviroid-like Element from Plants. Proc. Natl. Acad. Sci. USA.

[177] Lee B.D., Neri U., Roux S., Wolf Y.I., Camargo A.P., Krupovic M., Simmonds P., Kyrpides N., Gophna U., RNA Virus Discovery Consortium (2023). Mining Metatranscriptomes Reveals a Vast World of Viroid-like Circular RNAs. Cell.

[178] He S., Bing J., Zhong Y., Zheng X., Zhou Z., Wang Y., Hu J., Sun X. (2024). PlantCircRNA: A Comprehensive Database for Plant Circular RNAs. Nucleic Acids Res..

[179] Meyer K.D., Patil D.P., Zhou J., Zinoviev A., Skabkin M.A., Elemento O., Pestova T.V., Qian S.B., Jaffrey S.R. (2015). 5’ UTR m(6)A Promotes Cap-Independent Translation. Cell.

[180] Fan X., Yang Y., Chen C., Wang Z. (2022). Pervasive Translation of Circular RNAs Driven by Short IRES-like Elements. Nat. Commun..

[181] Diallo L.H., Tatin F., David F., Godet A.C., Zamora A., Prats A.C., Garmy-Susini B., Lacazette E. (2019). How Are CircRNAs Translated by Non-Canonical Initiation Mechanisms?. Biochimie.

[182] Chen R., Wang S.K., Belk J.A., Amaya L., Li Z., Cardenas A., Abe B.T., Chen C.K., Wender P.A., Chang H.Y. (2022). Engineering Circular RNA for Enhanced Protein Production. Nat. Biotechnol..

[183] López-Galiano M.J., Chiba S., Forgia M., Navarro B., Cervera A., Babaian A., Di Serio F., Turina M., de la Peña M. (2024). Self-Cleaving Ribozymes Conserved in RNA Viruses Unveil a New Role in Protein Translation. bioRxiv.

[184] Liu B., Qian S.B. (2014). Translational Reprogramming in Cellular Stress Response. Wiley Interdiscip. Rev. RNA.

[185] Ingolia N.T., Brar G.A., Stern-Ginossar N., Harris M.S., Talhouarne G.J.S., Jackson S.E., Wills M.R., Weissman J.S. (2014). Ribosome Profiling Reveals Pervasive Translation Outside of Annotated Protein-Coding Genes. Cell Rep..

[186] Edgar R.C., Taylor J., Lin V., Altman T., Barbera P., Meleshko D., Lohr D., Novakovsky G., Buchfink B., Al-Shayeb B. (2022). Petabase-Scale Sequence Alignment Catalyses Viral Discovery. Nature.

[187] Kuhn J.H., Botella L., de la Peña M., Vainio E.J., Krupovic M., Lee B.D., Navarro B., Sabanadzovic S., Simmonds P., Turina M. (2024). *Ambiviricota*, a Novel Ribovirian Phylum for Viruses with Viroid-like Properties. J. Virol..

[188] Forgia M., Navarro B., Daghino S., Cervera A., Gisel A., Perotto S., Aghayeva D.N., Akinyuwa M.F., Gobbi E., Zheludev I.N. (2023). Hybrids of RNA Viruses and Viroid-like Elements Replicate in Fungi. Nat. Commun..

[189] Kehr J., Kragler F. (2018). Long Distance RNA Movement. New Phytol..

[190] Ham B.K., Lucas W.J. (2017). Phloem-Mobile RNAs as Systemic Signaling Agents. Annu. Rev. Plant Biol..

[191] Kondhare K.R., Kumar A., Hannapel D.J., Banerjee A.K. (2018). Conservation of Polypyrimidine Tract Binding Proteins and Their Putative Target RNAs in Several Storage Root Crops. BMC Genom..

[192] Banerjee A.K., Chatterjee M., Yu Y., Suh S.G., Miller W.A., Hannapel D.J. (2006). Dynamics of a Mobile RNA of Potato Involved in a Long-Distance Signaling Pathway. Plant Cell.

[193] Sharma P., Lin T., Hannapel D.J. (2016). Targets of the StBEL5 Transcription Factor Include the FT Ortholog StSP6A1. Plant Physiol..

[194] Gopinath K., Kao C.C. (2007). Replication-Independent Long-Distance Trafficking by Viral RNAs in *Nicotiana benthamiana*. Plant Cell.

[195] Lezzhov A., Atabekova A., Tolstyko E., Lazareva E., Solovyev A. (2019). RNA Phloem Transport Mediated by Pre-MiRNA and Viral TRNA-like Structures. Plant Sci..

[196] Ding B. (2010). Viroids: Self-Replicating, Mobile, and Fast-Evolving Noncoding Regulatory RNAs. Wiley Interdisc. Rev. RNA.

[197] Zhang W., Thieme C.J., Kollwig G., Apelt F., Yang L., Winter N., Andresen N., Walther D., Kragler F. (2016). TRNA-Related Sequences Trigger Systemic MRNA Transport in Plants. Plant Cell.

[198] Thieme C.J., Rojas-Triana M., Stecyk E., Schudoma C., Zhang W., Yang L., Minãmbres M., Walther D., Schulze W.X., Paz-Ares J. (2015). Endogenous Arabidopsis Messenger RNAs Transported to Distant Tissues. Nat. Plants.

[199] Tolstyko E., Lezzhov A., Solovyev A. (2019). Identification of MiRNA Precursors in the Phloem of Cucurbita Maxima. PeerJ.

[200] Wang Y.H., Warren J.T. (2010). Mutations in Retrotransposon AtCOPIA4 Compromises Resistance to *Hyaloperonospora parasitica* in *Arabidopsis thaliana*. Genet. Mol. Biol..

[201] Zervudacki J., Yu A., Amesefe D., Wang J., Drouaud J., Navarro L., Deleris A. (2018). Transcriptional Control and Exploitation of an Immune-Responsive Family of Plant Retrotransposons. EMBO J..

[202] Ito H., Gaubert H., Bucher E., Mirouze M., Vaillant I., Paszkowski J. (2011). An SiRNA Pathway Prevents Transgenerational Retrotransposition in Plants Subjected to Stress. Nature.

[203] Lim C.J., Yang K.A., Hong J.K., Choi J.S., Yun D.J., Hong J.C., Chung W.S., Lee S.Y., Cho M.J., Lim C.O. (2006). Gene Expression Profiles During Heat Acclimation in Arabidopsis Thaliana Suspension-Culture Cells. J. Plant Res..

[204] Papolu P.K., Ramakrishnan M., Wei Q., Vinod K.K., Zou L.H., Yrjala K., Kalendar R., Zhou M. (2021). Long Terminal Repeats (LTR) and Transcription Factors Regulate PHRE1 and PHRE2 Activity in Moso Bamboo Under Heat Stress. BMC Plant Biol..

[205] Cho J., Paszkowski J. (2017). Regulation of Rice Root Development by a Retrotransposon Acting as a MicroRNA Sponge. Elife.

[206] McCue A.D., Nuthikattu S., Reeder S.H., Slotkin R.K. (2012). Gene Expression and Stress Response Mediated by the Epigenetic Regulation of a Transposable Element Small RNA. PLoS Genet..

[207] Weber C., Nover L., Fauth M. (2008). Plant Stress Granules and MRNA Processing Bodies Are Distinct from Heat Stress Granules. Plant J..

[208] Erickson S.L., Lykke-Andersen J. (2011). Cytoplasmic MRNP Granules at a Glance. J. Cell Sci..

[209] Zhang H., Tao Z., Hong H., Chen Z., Wu C., Li X., Xiao J., Wang S. (2016). Transposon-Derived Small RNA Is Responsible for Modified Function of WRKY45 Locus. Nat. Plants.

[210] Wang J., Zhou L., Shi H., Chern M., Yu H., Yi H., He M., Yin J., Zhu X., Li Y. (2018). A Single Transcription Factor Promotes Both Yield and Immunity in Rice. Science.

[211] Diener T.O. (1981). Are Viroids Escaped Introns?. Proc. Natl. Acad. Sci. USA.

[212] Martínez de Alba A.E., Flores R., Hernández C. (2002). Two Chloroplastic Viroids Induce the Accumulation of Small RNAs Associated with Posttranscriptional Gene Silencing. J. Virol..

[213] Zhou J., Yuan M., Zhao Y., Quan Q., Yu D., Yang H., Tang X., Xin X., Cai G., Qian Q. (2021). Efficient Deletion of Multiple Circle RNA Loci by CRISPR-Cas9 Reveals Os06circ02797 as a Putative Sponge for OsMIR408 in Rice. Plant Biotechnol. J..

[214] Marquez-Molins J., Hernandez-Azurdia A.G., Urrutia-Perez M., Pallas V., Gomez G. (2022). A Circular RNA Vector for Targeted Plant Gene Silencing Based on an Asymptomatic Viroid. Plant J..

[215] Senthil-Kumar M., Mysore K.S. (2014). Tobacco Rattle Virus-Based Virus-Induced Gene Silencing in *Nicotiana benthamiana*. Nat. Protoc..

[216] Ma J., Mudiyanselage S.D.D., Wang Y. (2022). Emerging Value of the Viroid Model in Molecular Biology and Beyond. Virus Res..

[217] Traber G.M., Yu A.-M. (2023). RNAi-Based Therapeutics and Novel RNA Bioengineering Technologies. J. Pharmacol. Exp. Ther..

[218] Daròs J.A., Aragonés V., Cordero T. (2018). A Viroid-Derived System to Produce Large Amounts of Recombinant RNA in *Escherichia coli*. Sci. Rep..

[219] Ortolá B., Cordero T., Hu X., Daròs J.A. (2021). Intron-Assisted, Viroid-Based Production of Insecticidal Circular Double-Stranded RNA in *Escherichia coli*. RNA Biol..

[220] Hu X., Richtman N.M., Zhao J.Z., Duncan K.E., Niu X., Procyk L.A., Oneal M.A., Kernodle B.M., Steimel J.P., Crane V.C. (2016). Discovery of Midgut Genes for the RNA Interference Control of Corn Rootworm. Sci. Rep..

[221] Puttaraju M., Been M. (1992). Group I Permuted Intron-Exon (PIE) Sequences Self-Splice to Produce Circular Exons. Nucleic Acids Res..

[222] Qu L., Yi Z., Shen Y., Lin L., Chen F., Xu Y., Wu Z., Tang H., Zhang X., Tian F. (2022). Circular RNA Vaccines Against SARS-CoV-2 and Emerging Variants. Cell.

[223] Zhang Z., Fu Y., Ju X., Zhang F., Zhang P., He M. (2024). Advances in Engineering Circular RNA Vaccines. Pathogens.

